# Hidden in Plain Sight: Integrative Taxonomy Discovers Two New Species of Digitate Soft Corals in the Urban Waters of China's Greater Bay Area

**DOI:** 10.1002/ece3.72228

**Published:** 2025-09-25

**Authors:** Yi‐Xuan Li, Janice Wingyan Ng, Haixin Loke, Lan Liu, Jian‐Wen Qiu

**Affiliations:** ^1^ Department of Biology Hong Kong Baptist University Hong Kong China; ^2^ School of Marine Sciences Sun Yat‐Sen University Zhuhai China; ^3^ Southern Marine Science and Engineering Guangdong Laboratory (Zhuhai) Zhuhai China

**Keywords:** Hong Kong, octocoral, phylogenetics, soft coral, South China Sea, taxonomy

## Abstract

Digitate or lobate scleralcyonacean soft corals are common in tropical reef ecosystems, yet their morphological plasticity and insufficient genetic information hinder our understanding of their diversity. In the China Seas, only four such species, all in the genus *Paraminabea* (Coralliidae), have been documented. Here, we describe *Parasphaerasclera dimorpha* sp. nov. (Parasphaerascleridae) and *Paraminabea inflata* sp. nov. from the urban waters of Hong Kong and Zhuhai. These species are distinct in morphology (colony shape and sclerite structure) and genetics from previously reported species. Genome skimming data of two new species and *Param. rubeusa* from two families were analyzed. Phylogenetic analyses were conducted using *MutS*, *28S* rRNA, and 14 mitochondrial protein‐coding genes, which recovered *Paras. dimorpha* sp. nov. as sister to *Paras. grayi* and *Param. inflata* sp. nov. as sister to *Param. aldersladei*. Seven mitochondrial gene orders and six rearrangement events were detected across families, and Parasphaerascleridae represents an early diverging clade within scleralcyonacean soft corals with a conserved gene order. In contrast, within Coralliidae, there are seven gene rearrangement patterns. Overall, we discovered two new species of digitate soft corals, underscoring the high diversity of soft corals in China's Hong Kong–Zhuhai–Macau Greater Bay Area and the urgency of documenting cryptic marine diversity in this highly urbanized area. Our genome skimming data for these two species, as well as a species without any molecular data before this study (i.e., *Param. rubeusa*), will be useful for further phylogenetic studies of soft corals.

## Graphical abstract

This article identified two novel soft coral species (*Parasphaerasclera dimorpha* sp. nov. and *Paraminabea inflata* sp. nov.) from urbanized South China Sea waters, distinct in morphology and genetics. Our study highlighted undocumented coral diversity in the hyper‐urbanized Greater Bay Area, emphasizing the need for cryptic species documentation in anthropogenically impacted ecosystems.

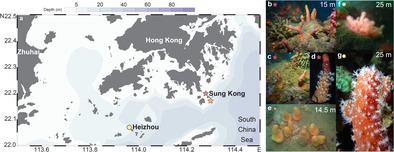

## 1 Introduction

Scleralcyonacean soft corals (Octocorallia: Scleralcyonacea) play crucial roles in marine benthic ecosystems as habitat engineers (Maucieri and Baum 2023), yet their taxonomy remains plagued by morphological convergence and insufficient molecular characterization (Bryce et al. 2015). This challenge is particularly pronounced in the azooxanthellate genera *Parasphaerasclera* McFadden and van Ofwegen, 2013 (Parasphaerascleridae McFadden and van Ofwegen, 2013) and *Paraminabea* Williams and Alderslade, 1999 (Coralliidae Lamouroux, 1812), which exhibit similar colony architectures despite distinct evolutionary trajectories (McFadden and van Ofwegen 2013). Of the recognized species in these genera (McFadden et al. 2025a, 2025b), genetic data are available for only six species of *Parasphaerasclera* and one of *Paraminabea* (i.e., *Param. aldersladei*)—a critical gap to the understanding of their phylogeny and biogeography (Quattrini et al. 2025).

*Parasphaerasclera* was established to accommodate species of *Eleutherobia* Pütter, 1900, and *Alcyonium* Linnaeus, 1758. Most *Parasphaerasclera* species are finger‐like with a bare stalk, comprising coenenchymal mounds when retracted, azooxanthellate without polyp sclerites, and sclerites predominantly radiate and tuberculate spheroids (McFadden and van Ofwegen 2013). However, *Parasphaerasclera* is often identified as *Paraminabea* due to a similar colony shape (McFadden and van Ofwegen 2013). *Paraminabea* was separated from *Minabea* Utinomi, 1957, with distinct spindle‐like and tuberculate spheroid sclerites. These *Paraminabea* species are azooxanthellate with long and white polyps. While some species from the two genera appear to exhibit endemism (e.g., *Paras. albiflora* in Sagami Bay), others like *Paras. grayi* and *Param. aldersladei* demonstrate trans‐oceanic distributions spanning Western Australia–Northwest Pacific (Benayahu et al. 2004; Bryce et al. 2015). However, inadequate sampling across their Indo‐Pacific ranges potentially masks cryptic diversity. These deficiencies not only hinder phylogenetic resolution within these Scleralcyonacea but also compromise ecological studies that are dependent on accurate species identification.

Hong Kong is situated on the southern coast of China. It borders the city of Shenzhen in Guangdong province, facing the Pearl River Estuary to the west and the South China Sea to the south and east. Thanks to its subtropical climate and complex coastal habitats, Hong Kong's relatively small marine area of 1640 km^2^ supports one of the most well‐developed coral communities in the northern South China Sea (Huang et al. 2015; Yeung et al. 2021; Zhao et al. 2022), and is home to 90 species of scleractinian corals (Chan et al. 2005; Yiu and Qiu 2022). Since the 1980s, studies have documented the decline of these urban coral communities, attributing losses to various factors such as reclamation and pollution (Wong et al. 2018), hypoxia (Binne Consultants Ltd 1995), coral bleaching (McCorry 2002; Xie et al. 2020; Chung et al. 2024), corallivory (Morton and Blackmore 2009), bioerosion (Lam et al. 2007; Dumount et al. 2013; Qiu et al. 2014; Xie et al. 2016), and recreational activities (Chung et al. 2013; Au et al. 2014).

Compared to the scleractinian corals, little is known about the soft corals. Most of our knowledge about the diversity and ecology of these species comes from several surveys and collections, which revealed a total of 29 species across 14 genera and 10 families in Scleralcyonacea and Malacalcyonacea (Zou and Scott 1982; Li 1986; Clark 1997; Fabricius and Alderslade 2001; Fabricius and McCorry 2006; Yeung et al. 2014) and found that environmental factors like turbidity and nutrients influence their distribution (Fabricius and McCorry 2006; Yeung et al. 2014). Among them, nine species from three families (i.e., Coralliidae, Ellisellidae Gray 1859, and Parisididae Aurivillius 1931) within Scleralcyonacea have been reported. Only three species (i.e., *Param. hongkongensis* Lam and Morton 2008; *Param. rubeusa* Benayahu and Fabricius 2010; *Param. indica* Thomson and Henderson 1905) are digitate or lobate soft corals. Like many other marine invertebrates in Hong Kong (Zhao et al. 2024), there is a big gap in DNA barcoding of soft corals (11 of 29 species with available barcodes), which hinders our understanding of genetic diversity and delineation of potential cryptic species.

**FIGURE 1:**
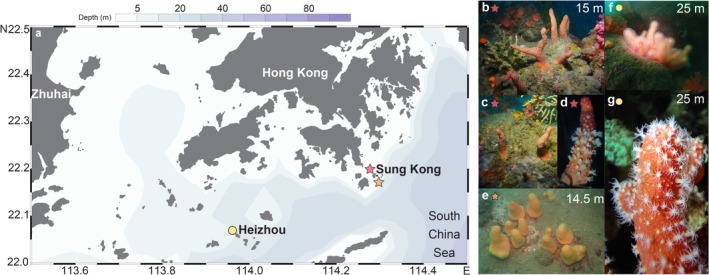
Map of sampling stations (a) and in situ photo of *Parasphaerasclera dimorpha* sp. nov. (b–d), *Paraminabea inflata* sp. nov. (e), and *Paraminabea rubeusa* (f, g).

During SCUBA diving in 2023 and 2024, we collected eight colonies of scleralcyonacean soft corals from the families Parasphaerascleridae and Coralliidae from Sung Kong, Hong Kong, and an adjacent area in Heizhou, Zhuhai (Figure 1). Our preliminary analysis indicated that these specimens belong to *Paraminabea rubeusa* Benayahu and Fabricius 2010, as well as to two undescribed species: one of *Parasphaerasclera* and the other of *Paraminabea*. Herein, we provide a morphological description of the two undescribed species and report the results of shallow‐level genome sequencing (i.e., genome skimming) to generate DNA sequences (i.e., mitochondrial genomes and *28S* rRNA) for the three species. These sequences are used to determine the placements of the three soft coral species within the phylogenetic framework of Scleralcyonacea and assess mitochondrial gene order rearrangement events among these corals. Our findings contribute to a better understanding of the diversity and genetics of these little‐known Scleralcyonacea.

## 2 Materials and Methods

### 2.1 Sample Collection

Samples were collected by SCUBA off Sung Kong (Hong Kong) and Heizhou Island (Zhuhai) at 14.5 to 25 m depths (Figure 1). Two colonies of *Parasphaerasclera dimorpha* sp. nov., five colonies of *Paraminabea inflata* sp. nov., and one colony of *Paraminabea rubeusa* were collected (Figure 1 and Figure S1). The holotype of *Paras. dimorpha* sp. nov. and *Param. inflata* sp. nov. were found on volcanic rocks in bottom waters surrounding Sung Kong Island, with an average water temperature of 23°C, an average salinity of 33.6 psu, and an average turbidity of 6.8 NTU during 2020–2024, according to records from the Environmental Protection Department (https://cd.epic.epd.gov.hk/EPICRIVER/vicmarineannual/result/). Samples were kept in seawater in the field and fixed in absolute ethanol in the laboratory.

### 2.2 Morphological Observation

Tissues from different parts of the colony were dissolved in 10% sodium hypochlorite and prepared following a procedure described in Williams and Mattison (2018). Dry sclerites were carefully placed on examine stubs, coated with gold, then observed with scanning electron microscopes: LEO 1530 Field Emission Scanning Electron Microscope (LEO Elektronenmikroskopie GmbH, Oberkochen, Germany) or Phenom Pro G6 Desktop SEM (ThermoFisher Scientific, USA).

In total, seven type specimens were deposited in the Tropical Marine Biodiversity Collections of the South China Sea (TMBC), Chinese Academy of Sciences, Guangzhou, China, including one holotype and one paratype of *Parasphaerasclera dimorpha* sp. nov. and one holotype and four paratypes of *Paraminabea inflata* sp. nov.

### 2.3 DNA Extraction and Sequencing

Genomic DNA was extracted from tissue samples of the three species using the QIAGEN DNeasy Tissue & Blood Kit (QIAGEN, Germany) following the manufacturer's protocol. The DNA quality was examined on a 1.0% agarose gel, and the quantity was measured using a NanoDrop 2000 Spectrophotometer (Thermo Fisher Scientific, USA). The samples were used to prepare DNA libraries and sequenced using the Novaseq 6000 platform in Novogene (Tianjin, China) to produce 150‐bp paired‐end reads.

### 2.4 Data Processing, Phylogenetics and Genetic Distance Analyses

Raw data were trimmed and filtered using Trimmomatic v.0.59 (Bolger et al. 2014), then assembled using SPAdes v.3.15.5 (Nurk et al. 2013) under the default settings. The mitochondrial genome, *MutS*, and *28S* genes of five specimens from the three species were extracted by BLAST search against the reference sequences from *Parasphaerasclera* and *Paraminabea*. The mitochondrial genomes were annotated using MITOS2 (Donath et al. 2019) in Galaxy Europe and Geseq (Tillich et al. 2017), and the sequences of each gene were further confirmed according to the results of BLASTn. The mitochondrial genomes were plotted to show their structural features using Chloroplot (Zheng et al. 2020).

Phylogenetic analyses were conducted to determine the phylogenetic placement of the three species within Scleralcyonacea using the *MutS* gene, the *28S* rRNA gene, and the mitochondrial genome data. The *MutS* dataset (2949 bp) and *28S* dataset (795 bp) were prepared using sequences available in public databases for seven *Parasphaerasclera* and three *Paraminabea* species and one outgroup, *Alcyonium digitatum*. The mitochondrial genomic dataset (14‐mt‐gene matrix, 16726 bp) comprised 14 protein‐coding genes (PCGs) of the mitochondrial genome of the three species recovered in this study and 75 other species of Scleralcyonacea (except for sea pens) available in public datasets. For each data matrix, phylogenetic analyses were conducted using PhyloSuite v.1.2.3 (Zhang et al. 2020) with several plugins: (1) applied Mafft v. 7.505 (Katoh et al. 2002) and trimAL v.1.2 (Capella‐Gutiérrez et al. 2009) with default settings and “‐‐automated” to align and trim the dataset; (2) then, we used Modelfinder v.1.5.4 (Kalyaanamoorthy et al. 2017) embedded in IQ‐TREE2 v.2.2.0 (Minh et al. 2020) to select the best‐fit model; (3) the Maximum Likelihood (ML) analyses were performed using IQ‐TREE2 under the edge‐unlinked partition model for 10,000 ultrafast bootstraps (Hoang et al. 2018), as well as the Shimodaira–Hasegawa–like approximate likelihood‐ratio test (Guindon et al. 2010); (4) while Bayesian inference (BI) analyses for three matrices were performed using MrBayes v.3.2.7a (Ronquist et al. 2012) in two runs with 1,000,000 MCMC iterations, sampling every 100 iterations, 25% burn‐in. The output of MrBayes was examined by Tracer v.1.7.2 (Rambaut et al. 2018) with the ESS value over 250. The phylogenetic trees were plotted by iTOL v.5 (Letunic and Bork 2021). Information on data matrices and the best‐fit model for each matrix is included in Table S1.

To determine the genetic divergence between species, we calculated the Kimura 2‐parameter (K2P) genetic distance within *Parasphaerasclera* and *Paraminabea* and between outgroups using a reduced *MutS* matrix (573 bp, all gaps removed) using MEGA 11 (Tamura et al. 2021) based on 1000 bootstrap replications. *MutS* is a genetic marker that has been widely used in species delimitation of soft corals (Quattrini et al. 2019). Species delimitation analysis using the *MutS* matrix was conducted using ASAP v.1.0 (Puillandre et al. 2020) with the K2P model and default settings.

Gene order rearrangement events for soft coral families (summarized topology from 14‐mt‐gene matrix) were detected using TreeRex v.1.8 (Bernt and Middendorf 2008) based on the common interval algorithm.

## 3 Results

### 3.1 Phylogenetic Relationships and Species Delimitation Based on Single Genes

**FIGURE 2:**
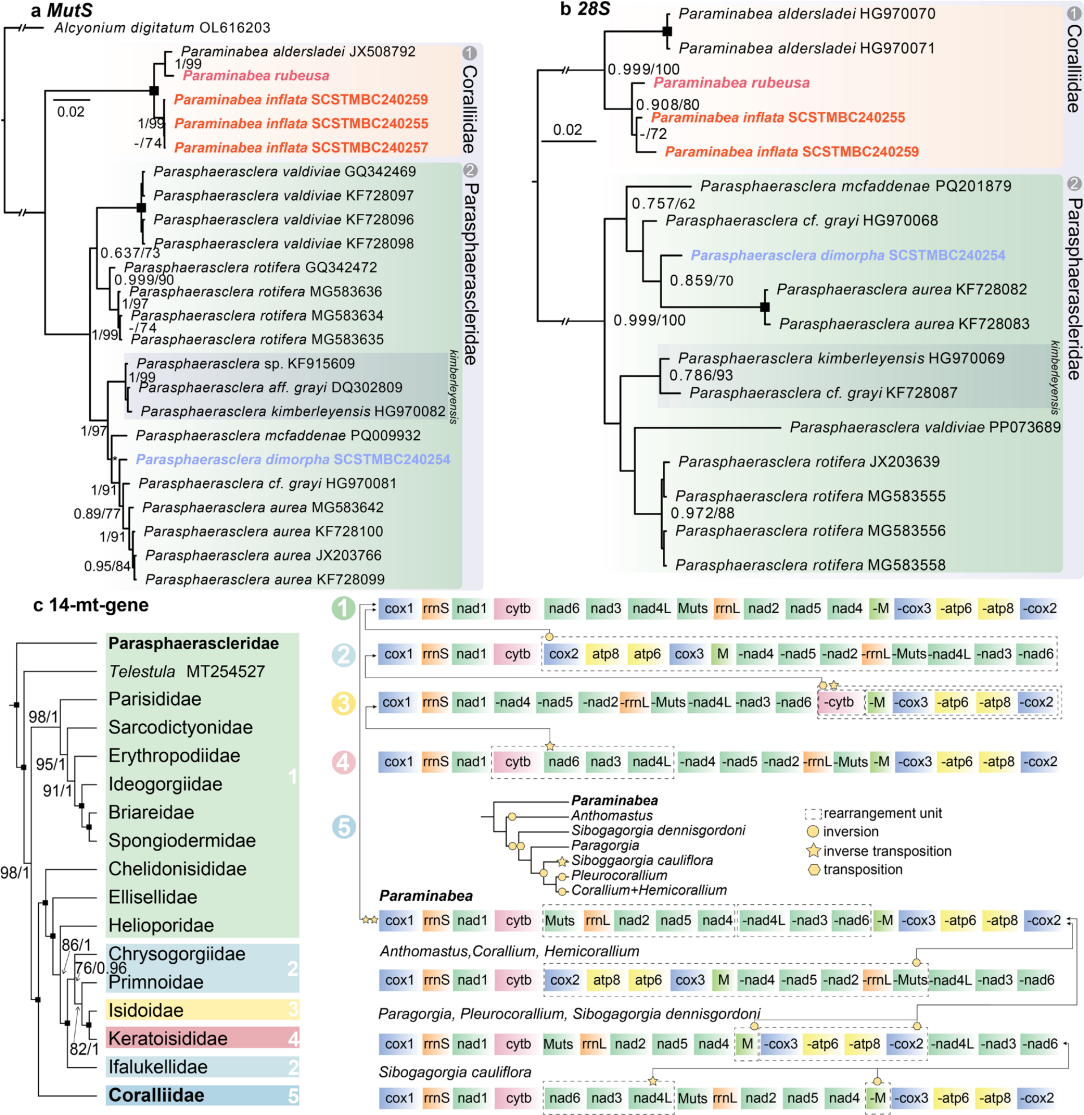
Phylogenetic relationships of *Parashaerasclera* and *Paraminabea*. The phylogenetic results using Bayesian Inference analysis are based on *MutS* (a), *28S* (b) gene fragments, and 14 protein‐coding genes (c), separately. Square in node, fully supported by ML and BI analysis; Value in node, Ultrafast Bootstrap value (UFB)/Bayesian Posterior Probability (BPP) over 70/0.70; “‐” or blank, node support value lower than 70/0.70; * in node, conflict placement between ML and BI approaches.

Molecular phylogenetic (Figure 2a,b) and genetic distance (Table 2) analyses were conducted to delimit species and determine the relationships between the new species and other *Paraminabea* and *Parasphaerasclera* soft corals. Unfortunately, only *Param. aldersladei* has the corresponding molecular data before this study among the ten described *Paraminabea* species. Our phylogenetic analyses based on single genes (*MutS* and *28S*, Figure 2a,b) showed that *Param. inflata* sp. nov. is distant from *Param. rubeusa* and *Param. aldersladei*. Based on the *MutS* gene, K2P genetic distances between *Paraminabea* species are all < 2.0% (Table 2), that is, 1.24% divergence between *Param. inflata* and *Param. aldersladei* and 1.77% divergence between *Param. inflata* and *Param. rubeusa*. Nevertheless, these values are much larger than the 0.88% divergence between *Param. aldersladei* and *Param. rubeusa*, supporting the recognition of *Param. inflata* as a new species.

Among the ten recognized species of *Parasphaerasclera*, molecular data for seven species were available before this study. However, the specimens of *Paras*. aff. *grayi* and *Paras. grayi* used for sequencing the *MutS* gene were placed in different clades (Figure 2a), *Paras.* aff. *grayi* (GenBank accession number DQ302809) as sister species with *Paras. kimberleyensis* and the latter (GenBank accession number HG970081) as sister species for *Paras. aurea*. Moreover, the two sequences had a K2P distance of 2.0%, which is higher than the interspecific distances between other species in this genus, that is, 1.24% divergence between *Paras. dimorpha* and *Paras. aurea* (Table S2). Similarly, in the *28S* tree (Figure 2b), one (accession number HG970068) was sister to *Paras. aurea* and *Paras. dimorpha*, and another (accession number KF728087) formed a clade with *Paras. kimberleyensis*. Indeed, the individual corresponding to accession number DQ302809 and *Parasphaerasclera* sp. were identical to *Paras. kimberleyensis* when all gaps in the *MutS* alignment were removed (Table 2), congruent with the species delimitation result (Figure S2). For the *MutS* tree (Figure 2a), we recovered two major clades, (*valdiviae* + *rotifera*) and (*kimberleyensis* + (*mcfaddenae* + (*hongkongensis* + (*grayi* + *aurea*)))) using BI analysis. The placement of *Paras. mcfaddenae* is not stable due to conflicts between the ML and BI results. Nevertheless, *Paras. dimorpha* was recovered as sister to *Paras. grayi* and *Paras. aurea* in the *MutS* and *28S* trees (Figure 2a,b). After combining the three identical sequences of *Paras. kimberleyensis*, *Paras. dimorpha* sp. nov. was calculated to show a 0.88% divergence with *Paras. grayi* and *Paras. aurea*, but *Paras. grayi* was more closely related to *Paras. aurea* than our new species with a 0.70% divergence (Table 2), supporting *Paras. dimorpha* sp. nov. as a distinct species.

### 3.2 Phylogenetic Relationships and Gene Orders Revealed by Mitochondrial Genomes

The complete mitochondrial genomes of the three species (Figure S3) range from 18,301 bp to 19,997 bp in length, with negative AT skew and positive GC skew (Table S3). The phylogenetic relationships of Scleralcyonacea soft coral families, constructed with available mitochondrial genomes, were assessed, and the gene rearrangement patterns were examined (Figure 2c, Figure S4). Phylogenetic analyses showed that Parasphaerascleridae represents the earliest‐diverging clade of Scleralcyonacea, which is sister to a clade comprising all other families of this order (Figure 2c, Figure S4). Within this big clade that is sister to Paraphaerascleridae, *Telestula* MT254527 is sister to a clade including six families (i.e., Parisididae + (Sarcodictyonidae McFadden, van Ofwegen and Quattrini, 2024 + (Erythropodiidae Kükenthal, 1916 + (Ideogorgiidae McFadden, van Ofwegen and Quattrini, 2024 + (Briareidae Gray, 1859 + Spongiodermidae Wright and Studer, 1889))))) and a clade comprising Coralliidae and the rest of the families. The two clades were split into 15 families with high supporting values (UFB > 95, BPP > 0.95). The familiar status of *Telestula* MT254527 requires further investigation.

There were seven gene arrangement types among the available mitochondrial genomes of soft corals (Figure 2c). Integrating the gene‐order information into the phylogenetic tree, it is clear that the mitochondrial gene order represented by Parasphaerascleridae (i.e., GO1) is the ancestral state for the order Scleralcyonacea. The gene order changes align well with the relationship revealed by the topology at the family level. Most soft coral families (10/16) exhibited an identical gene arrangement with Parasphaerascleridae (GO1). The three *Parasphaerasclera* mitochondrial genomes exhibit an identical gene order (GO1). The Chrysogorgiidae Verrill, 1883, Primnoidae Milne Edwards, 1857, and Ifalukellidae Bayer, 1955 share the same gene order (GO2), while Isidoidae (GO3) and Keratoisididae Gray, 1870 (GO4) exhibit another two gene arrangements. The gene block comprising *cox1*‐*rrnS*‐*nad1* is conserved for all gene order types, and rearrangements were observed for the other 14 genes. GO2 has an inversion for the gene block containing 13 genes compared to GO1 (Figure 2c). Then, the clade of Isidoidae and Keratoisididae both exhibit three rearrangement events compared to GO2, including transposition of *cytb* and gene block *trnM*‐*cox3*‐*atp6*‐*atp8*‐*cox2* and inverse transposition of gene block *cytb*‐*trnM*‐*cox3*‐*atp6*‐*atp8*‐*cox2*. Differing from GO3, Keratoisididae (GO4) further exhibits an inverse transposition change of gene block *cytb*‐*nad6*‐*nad3*‐*nad4L*. Among these soft coral families, Coralliidae species exhibit complex rearrangement changes (GO5). *Paraminabea*, representing the first splitting clade of Coralliidae, shows two inverse transpositions of the gene block *MutS*‐*rrnL*‐*nad2*‐*nad5* and *nad4L*‐*nad3*‐*nad6*, compared to GO1. Four gene arrangement types are identified for seven genera within the family Coralliidae (GO5). In contrast with *Paraminabea*, seven rearrangement events occurred that align with their phylogenetic relationships.

### 3.3 Systematics

**Family Parasphaerascleridae McFadden and Ofwegen**, 2013

**Genus Parasphaerasclera McFadden and Ofwegen**, 2013

**Parasphaerasclera dimorpha sp. nov**.

**FIGURE 3:**
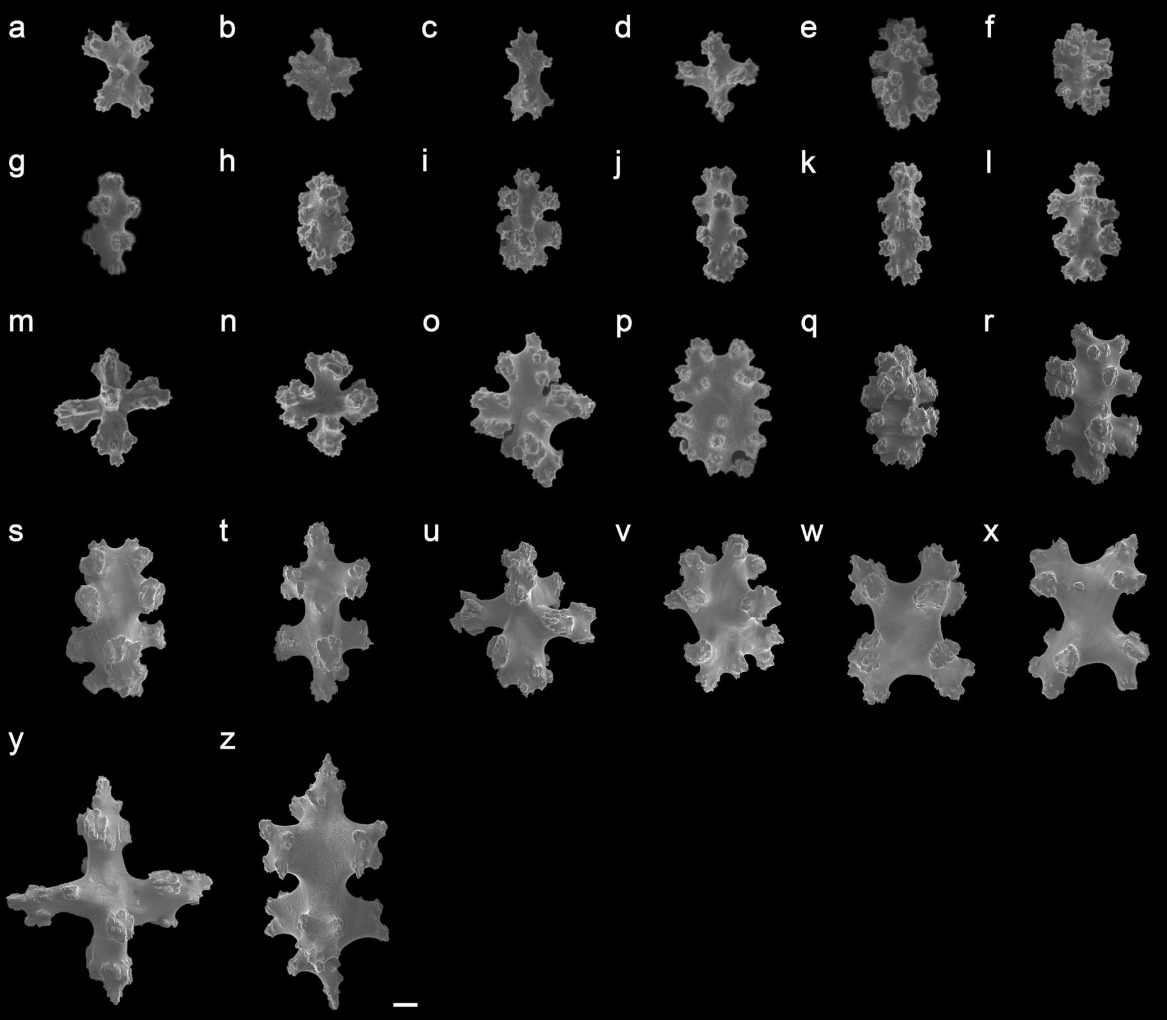
Sclerites of *Parasphaesclera dimorpha* sp. nov. holotype (SCSTMBC240253). (a–p) polyparium sclerites; (q–z) stalk sclerites; Scale bar, 10 μm.

**Zoobank:** https://zoobank.org/NomenclaturalActs/aff0bc03‐f528‐49fa‐bfbc‐ca45ed3cd816

(Figures 1b–d and 3, Figures S1 and S5)

3.3.1

##### Material Examined

SCSTMBC240253 (holotype) and SCSTMBC240254 (paratype), two specimens, east of Sung Kong Island, Hong Kong, 22.193850° N, 114.280363° E, SCUBA, depth 15 m, coll. 12 June 2024 by Haixin Loke.

##### Diagnosis

Digitiform or branched, light red orange, 70 mm in height and 20 mm in diameter (holotype SCSTMBC240253, size shrunk after collection), autozooid short with eight tentacles. No sclerites in polyps.

##### Type Locality

Sung Kong, 15 m, Hong Kong, China.

##### Description

Colony with digitate or branched growth forms, with a wide and bare stalk (Figure 1a). Size variable among colonies but most with a similar digitate shape, 65–70 mm in length and 18–20 mm in diameter for the two digitate colonies collected. In alcohol, size shrank dramatically by approximately half. Base without polyps, short. Polyps translucent, monomorphic, retractile, producing yellow coenenchymal mounds when retracted. Height of coenenchymal mounds similar to length of tentacles when fully extended (Figure 1 and Figure S1d). Eight tentacles, each with 6–9 pinnules arranged in two rows along each edge.

Polyp sclerites absent. All polyparium and stalk sclerites with irregular tubercles. For polyparium with coenenchymal mounds, the majority of sclerites (Figure 3a–p and Figure S5a–x) are tuberculate capstans, some slightly club‐shaped, some schistose‐shaped, along with crosses and a few rodlets. Polyparium sclerites are 0.035–0.073 mm in height and 0.020–0.067 mm in diameter (Figure 3a–p and Figure S3a–r). In stalk surface tissues, sclerites are spindles, clubs, crosses, and irregular forms, 0.052–0.106 mm in height and 0.032–0.086 mm in diameter (Figure 3q–z and Figure S5s–z).

##### Color

In situ, colonies light orange‐red color with small, bright, yellow rounded coenenchymal mounds. Polyp‐free encrusting base carrot color. Polyps including tentacles milky white and translucent. Colonies rusty red color after preservation in alcohol. Sclerites orange color.

##### Habitat

Scattered on boulders that were also populated with sun corals and a species of *Dendronephthya* sp.

##### Etymology

Named for its two colony forms observed.

##### Distribution

This species is only known from eastern Hong Kong waters.

##### Remarks

Within *Parasphaerasclera*, ten valid species (before this study) are distributed in three biogeographic regions: South Africa (*Paras. aurea*, *Paras. morifera*, *Paras. rotifera*, *Paras. valdivae*), Western Australia and Gulf of Mexico (*Paras. grayi*, *Paras. kimberleyensis*, and *Paras. mcfaddenae*), and the West Pacific Ocean (*Paras. albiflora*, *Paras. grayi*, *Paras. nezdoliyi*, *Paras. zanahoria*) (Quattrini et al. 2025). Notably, although most observations of *Paras. grayi* were in Western Australia, Benayahu et al. (2004) reported this species from southern Taiwan waters, East China Sea, closer to the locality of our new species than other species. *Parasphaerasclera dimorpha* sp. nov. is the only other species of this genus reported from the China Seas and exhibits dual morphological affinity (digitate or branched form). Compared to other congeneric species, *Paras. dimorpha* sp. nov. resembles more to *Paras. grayi* in being digitiform with a wide base. But the two species can be easily distinguished (Bryce et al. 2015) by (1) color: light orange‐red in *Paras. dimorpha* and bright rusty orange in *Paras. grayi* (Figure S6a,b); (2) growth form: digitate or branched in *Paras. dimorpha* and holdfast (twin digitates with a jointed base) in *Paras. grayi* (Figure S6a,b); and (3) sclerite morphology: stalk sclerites include spindles in *Paras. dimorpha* (Figure S6c) and rods with a long smooth shaft in *Paras. grayi* (Figure S6d).

**Family Coralliidae Lamouroux, 1812**

**Genus Paraminabea Williams and Alderslade**, 1999

**Paraminabea inflata sp. nov**.

**FIGURE 4:**
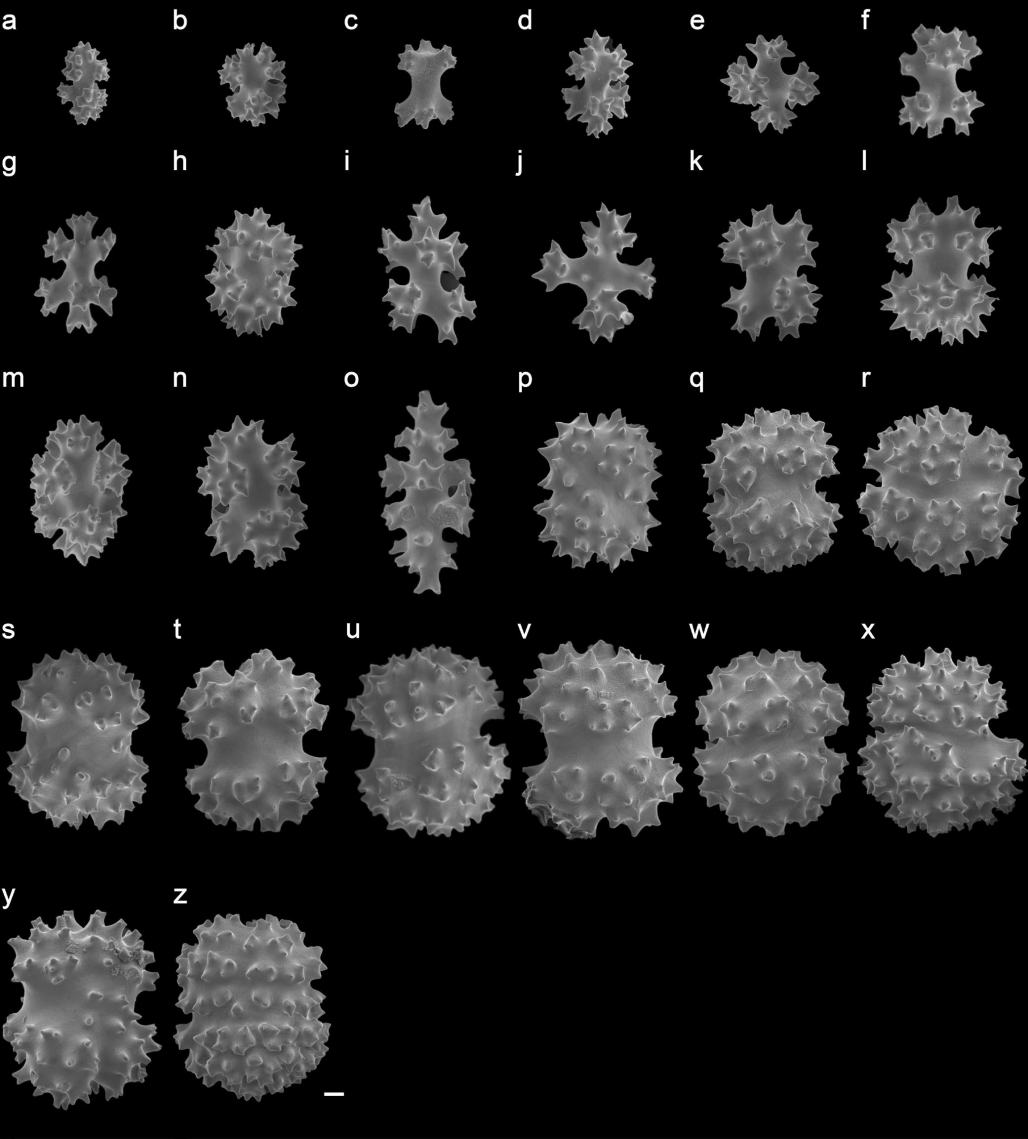
Sclerites of *Paraminabea inflata* sp. nov. holotype SCSTMBC240255. (a–o) polyparium sclerites; (p–z) stalk sclerites. Scale bar, 10 μm.

**Zoobank:** https://zoobank.org/NomenclaturalActs/d4bc7211‐db52‐413d‐b9b6‐098a5bc088af

(Figures 1e and 4, Figures S1, S7–S9)

##### Material Examined

SCSTMBC240255‐SCSTMBC240259, five specimens, around Sung Kong Island, Hong Kong (SCSTMBC240255, holotype, SCSTMBC240256‐SCSTMBC240258, paratypes), and Heizhou Island, Zhuhai (SCSTMBC240259, paratype), SCUBA, depth 14.5–25 m, coll. September 2023, May and June 2024 by Sam Yiu, Haixin Loke, and Lan Liu.

##### Diagnosis

Small, dome‐shaped or digitiform, sometimes twin colonies jointed at the base, yellow or orange colonies, 9 to 24 mm in diameter and 9 to 46 mm in height. Dimorphic polyps are yellow and white. Autozooids are long with slim bodies. No sclerites are in the polyps.

##### Type Locality

Sung Kong Island, 14.5 m, Hong Kong, China.

##### Description

Colony (Figure S1a,b) with dome‐shaped (SCSTMBC240256), digitate (SCSTMBC240255 and SCSTMBC240259), or domes in pairs linking ~2.5 mm spreading base (SCSTMBC240257), or dome‐lobate with linking ~0.5 mm spreading base (SCSTMBC240258). Size of single colonies 9–46 mm in height and 9–24 mm in width. Size of paired domes (SCSTMBC240257), the small dome 9 mm in diameter and 9 mm in height, and the large dome 15 mm in diameter and 29 mm in height. Polyp‐free basal portion short. No obvious color and size changes after preservation in alcohol. Autozooids with a long, slender body. When retracted, coenenchymal mounds yellow.

Polyp sclerites absent. Polyparium with coenenchymal mounds (Figure 4a–o, Figures S7a–p, S8a–n, and S9a–m), sclerites mostly barrels with six, seven, and eight radiates, double heads with median waist, spindles with elongated waist, and some in cruciform with six radiates. Size of polyparium sclerites variable, 0.041 to 0.106 mm in height and 0.027 to 0.090 mm in diameter. Stalk sclerites (Figure 4p–z, Figures S7q–z, S8o–v, and S9n–w) mostly sub‐spheroidal forms of similar size with a very short and thick waist (sometimes barely invisible), irregular forms with small radiates on surface, robust barrels, some with double heads with median waist (SCSTMBC240256), or cruciform with six radiates (SCSTMBC240256). Stalk sclerites 0.051 to 0.115 mm in height and 0.049 mm to 0.100 mm in diameter.

##### Color

Living and preserved colonies are yellow and orange in color; expanded autozooids are white but observed with a yellow base when retracted (Figure 1). Polyp‐free base is red or yellow. Polyps are transparent with white tentacles. Sclerites are peachy in color.

*Habitats*. Sparsely distributed or clustered, with bases attached to rock surfaces.

##### Etymology

Named for its colony shape, like an inflated sponge earplug.

##### Distribution

This species is currently known from Hong Kong and Zhuhai waters only. The iNaturalist website has 14 records of this species from Eastern (Conic Island) to Southern Hong Kong (Lamma Island), but it was incorrectly identified as *Paraminabea hongkongensis* (https://www.inaturalist.org/taxa/1376397‐Paraminabea‐hongkongensis, visited on 20 May 2025).

##### Remarks

There were ten extant species of *Paraminabea* before this study. According to HKRMS (https://www.marinespecies.org/hkrms/aphia.php?p=taxdetails&id=267691), three species have been recorded from Hong Kong, including *Param. indica*, *Param. hongkongensis*, and *Param. rubeusa*. Among them, *Param. rubeusa* differs from the other three species in its branching growing form and different sclerite shapes, such as elongated spindle‐like sclerites with thorns (Benayahu and Fabricius 2010). The same sclerite shape, cruciform with six rays, was observed in the polyparium and stalk of *Param. inflata* sp. nov. (Figure 4), *Param. rubeusa* (Figure S10), and *Param. hongkongensis* (Lam and Morton 2008). Such sclerites have been reported to be absent from other *Paraminabea* species (Lam and Morton 2008; Benayahu and Fabricius 2010). Although *Param. inflata* shares similar sclerite shapes with *Param. hongkongensis*, *Param. hongkongensis* lacks two types of sclerites found in *Param. inflata* sp. nov.: spindle‐like sclerites in the polyparium and sub‐spheroidal sclerites with a short waist in the stalk (Figure 4). The colony size of *Param. inflata* sp. nov. ranges from 9 mm to 46 mm, while *Param. hongkongensis*, discovered in a cave, was approximately 20 mm (Lam and Morton 2008). Further examination of *Param. hongkongensis* is needed to clarify the significance of size differences. In addition, *Param. indica* (type locality in the Indian Ocean) lacks the sclerite shape of tuberculated spheroids and cruciform sclerites with six rays, and its polyps are only distributed in half of the colony. *Param. aldersladei* (Bryce et al. 2015) reassembles *Param. inflata* sp. nov. in possessing tuberculated spheroids in the polyparium and stalk, but we only observed such sclerites in the stalk of *Param. inflata* sp. nov. with a thicker waist. Besides, *Param. aldersladei* can be distinguished from *Param. inflata* sp. nov. by polyp shape (completely retracted autozooid) and sclerite distributions (similar sclerite shapes were observed in both the polyparium and stalk base).

## 4 Discussion

Our integrative morphological and genetic analyses support the recognition of *Paras. dimorpha* sp. nov. and *Param. inflata* sp. nov. *Paraminabea* and *Parasphaerasclera* are known for their confounding phenotype variability, and their identification relies heavily on sclerite morphology and molecular data (Bryce et al. 2015). *Param. inflata* sp. nov. colonies were historically misidentified as *Param. hongkongensis* (https://www.inaturalist.org/taxa/1376397‐Paraminabea‐hongkongensis) and were found alongside *Paras. rubeusa* (iNaturalist observation, https://www.inaturalist.org/observations/243070161, visited on 20 May 2025). Similarly, *Paras. dimorpha* sp. nov. was misidentified as *Param. hongkongensis* before this study (iNaturalist observations: https://www.inaturalist.org/observations/230916255, visited on 20 May 2025), and its colonies co‐occurred with *Param. inflata* sp. nov. at Sung Kong (our records, Figure 1 and Table 1) and East Tathong Point (iNaturalist observations: https://www.inaturalist.org/observations/230916255 and https://www.inaturalist.org/observations/230916739, visited on 20 May 2025).

**TABLE 1:** Sampling information.

Species	Voucher ID	Sampling site	Depth (m)	Coordinates	Collection date
*Parasphaerasclera dimorpha* sp. nov.	SCSTMBC240253^a^	Northwest Sung Kong, Hong Kong, China	15	22.194° N, 114.280° E	June 2024
	SCSTMBC240254^b^	Northwest Sung Kong, Hong Kong, China	15	22.194° N, 114.280° E	June 2024
*Paraminabea inflata* sp. nov.	SCSTMBC240255^a^	East Sung Kong, Hong Kong, China	14.5	22.185° N, 114.289° E	September 2023
	SCSTMBC240256^b^	East Sung Kong, Hong Kong, China	14.5	22.185° N, 114.289° E	June 2024
	SCSTMBC240257^b^	East Sung Kong, Hong Kong, China	14.5	22.184° N, 114.289° E	June 2024
	SCSTMBC240258^b^	East Sung Kong, Hong Kong, China	14.5	22.184° N, 114.289° E	June 2024
	SCSTMBC240259^b^	Heizhou, Zhuhai, China	25	22.061° N, 113.978° E	May 2024
*Paraminabea rubeusa*	N/A	Heizhou, Zhuhai, China	25	22.061° N, 113.978° E	May 2024

Abbreviation: N/A, not applicable.

^a^ Holotype.

^b^ Paratype.

The family Parasphaerascleridae is small, with 11 species (McFadden and van Ofwegen 2013; Bryce et al. 2015, this study). These species typically inhabit depths ranging from ~10 m (*Paras. kinberleyensis*) to 85 m (*Paras. mcfaddenae*), and some form populations in caves (Quattrini et al. 2025). Most of the species exhibit a single growth form (either digitiform or lobate), except for three species showing morphological plasticity—Paras. *dimorpha* sp. nov., *Paras. valdivae*, and *Paras. rotifera—*which possess an additional form of conspicuous stalks with branches. These three species can be distinguished by polyp retractility, sclerite shape, and geographic distribution. *Paras. grayi*, a morphologically variable species with a wide distribution (Indo‐West Pacific and Western Australia), resembles *Paras. dimorpha* sp. nov. more than others, particularly in its digitiform shape and proximity (southern edge of Taiwan). Notably, the orange coloration (Figure 1 and Figure S1) and spindle‐shaped sclerites in the stalk (Figure 3) of *Paras. dimorpha* sp. nov., combined with a 0.88% *MutS* divergence, differentiate it from *Paras. grayi* morphologically and genetically. Furthermore, *Paras. dimorpha* sp. nov. is the second species observed with tuberculated spindles, a trait it shares with *Paras. mcfaddenae*, though these spindles are shorter and less common in the former species (Figure 3).

Although *Paras. dimorpha* sp. nov. and *Param. inflata* sp. nov. are similar in colony shape, their stalk size, sclerite morphology, and genetic profiles are markedly distinct. Both *Param. rubeusa* and *Param. inflata* inhabit rock surfaces in deeper waters, extending to 25 m (Figure 1), but their colony shape, color, and sclerite morphology are distinct. Two other *Paraminabea* species in Hong Kong, *Param. hongkongensis* and *Param. indica*, differ from *Param. inflata* sp. nov. in the colony and stalk sclerite morphology. Among *Paraminabea* species, the tuberculated spheroids in the stalk of *Param. inflata* sp. nov. resemble those of *Param. aldersladei* (Bryce et al. 2015), though the distribution of their spheroids and their polyp shapes differ. Unfortunately, the lack of genetic data for most *Paraminabea* species hinders phylogenetic reconstruction, with only three species with available *MutS sequences* showing 0.88% to 1.77% divergences (Table 2). These two new species inhabit deep coastal waters (> 10 m) with low visibility and high‐frequency water exchange, likely adapting to high turbidity for enhanced food acquisition via filter‐feeding on suspended particles. Ecologically, as habitat engineers, they create complex structures that support benthic biodiversity and regulate sediment dynamics (Fabricius and Alderslade 2001). Besides, as biochemical reservoirs, their secondary metabolites may be a source of new biochemicals as their relatives (Chao et al. 2013).

**TABLE 2:** K2P distance analysis based on *MutS* gene fragment.

	1	2	3	4	5	6	7	8	9	10	11
*A. digitatum*	NA										
*Paras. valdiviae*	12.93	0.00									
*Paras. rotifera*	12.40	4.26	0.00								
*Paras. kimberleyensis*	12.27	4.36	3.15	**0.00**							
*Paras. grayi*	12.91	4.35	3.51	1.77	NA						
***Paras. dimorpha*** **sp.** **nov.**	12.69	4.54	3.33	1.59	**0.88**	NA					
*Paras. aurea*	13.36	4.64	3.51	1.77	**0.70**	**0.88**	0.00				
*Paras_mcfaddenae*	13.14	4.54	2.96	1.23	1.59	1.41	1.59	NA			
*Param. aldersladei*	15.37	8.62	7.52	7.43	7.42	8.02	7.82	7.22	NA		
***Param. rubeusa***	16.05	9.64	8.52	8.43	8.42	9.03	8.83	8.22	**0.88**	NA	
***Param. inflata*** **sp. nov.**	14.23	7.61	6.53	6.44	6.43	7.02	6.83	6.63	**1.24**	**1.77**	0.00

*Note:* A, *Alcyonium*; NA, not applicable due to fewer than three individuals within the group; Param, *Paraminabea*; Paras, *Parasphaerasclera*. Bold indicate new data provided in this study.

Our study not only revealed morphological differences between *Paras. dimorpha* sp. nov. and *Param. inflata* sp. nov., but also generated DNA sequences allowing for the determination of their placements in the phylogenetic tree of Scleralcyonacea, providing a broader understanding of the convergent evolution of the digitate growth form. The placement of Parasphaerascleridae within Scleralcyonacea was inconsistent across nuclear and mitochondrial loci in previous studies (Quattrini et al. 2023, 2024, 2025). Despite the low substitution rate of mitochondrial genes (McFadden et al. 2006), our data matrix with 14 protein‐coding genes (PCGs) from 78 species supported the distinction of the Parasphaerascleridae clade from other families (Figure 2c and Figure S4), which is congruent with the gene order results. That contrasted with the two‐clade topology of Scleralcyonacea using *MutS* (Quattrini et al. 2025), including one consisting of Parasphaerascleridae and Coralliidae. *MutS* is a powerful genetic marker for diagnosing soft coral species in this study and previous studies (Bryce et al. 2015; Quattrini et al. 2025). However, the insufficiently informative sites of *MutS* or the whole mitochondrial genome limit their resolution on a large scale, requiring large quantities of markers (i.e., Ultraconserved elements) from omics data to reconstruct reliable phylogenetic relationships within Scleralcyonacea soft corals (Quattrini et al. 2024). With robust family‐level topology support (Figure 2c and Figure S4), we explored the phylogenetic pattern of gene rearrangement events, particularly within Coralliidae. Seven gene orders were identified across 16 Scleralcyonacea soft coral families (Figure 2c), with only the “*cox1*‐*rrnS*‐*nad1*” block conserved among them, indicating a complex evolutionary pattern in these soft corals, especially within Coralliidae (Brockman and McFadden 2012; Quattrini et al. 2023).

## 5 Conclusion

We described *Parasphaerasclera dimorpha* sp. nov. and *Paraminabea inflata* sp. nov., supported by robust morphological and molecular evidence. This work enhances our knowledge of the biodiversity of these two little‐known genera of mainly digitiform or lobular soft corals. Notably, *Paras. dimorpha* is the first species of Parasphaerascleridae reported from the China Seas. For the three species studied, including *Param. rubeusa*, we provide the first set of DNA sequences, which allowed us to determine their phylogenetic positions. Our assembly of the mitochondrial genomes of the three species provides novel data for a better analysis of the gene order rearrangements within Scleralcyonacea.

## Author Contributions

**Yi‐Xuan Li:** data curation (lead), methodology (lead), visualization (lead), writing – original draft (lead), writing – review and editing (equal). **Janice Wingyan Ng:** data curation (equal), methodology (equal), visualization (equal), writing – review and editing (supporting). **Haixin Loke:** resources (lead), writing – review and editing (supporting). **Lan Liu:** resources (equal), writing – review and editing (supporting). **Jian‐Wen Qiu:** conceptualization (lead), funding acquisition (lead), project administration (lead), supervision (lead), writing – review and editing (equal).

## Conflicts of Interest

The authors declare no conflicts of interest.

## Supporting information


**Table S1:** Data matrices and analysis approaches.
**Table S2:** K2P distance analysis based on *MutS* gene fragment.
**Figure S1:** Colonies of *Paraminabea inflata* sp. nov. (a, b) and *Parasphaerasclera dimorpha* sp. nov. (c, d). Scale bar, 10 mm.
**Figure S2:** ASAP group results based on *MutS* dataset.
**Figure S3:** Mitochondrial genome architecture of three soft corals.
**Figure S4:** Phylogenetic analysis of soft corals using 14 mitochondrial coding genes (16,726 bp) and their gene order structure. This topology is supported by the ML analysis result. Square in node, fully supported by ML and BI analysis; Value in node, ultrabootstrap value/Bayesian Posterior Probability over 70/0.70; Ms.: *MutS*.
**Figure S5:** Polyparium (a‐r) and stalk (s–z) sclerites of *Parasphaesclera dimorpha* sp. nov. paratype (SCSTMBC240254). Scale bar, 10 μm.
**Figure S6:** Comparative morphological characteristics of *Parasphaesclera dimorpha* sp. nov. (a, c) and *Parasphaesclera grayi* (b, d).
**Figure S7:** Polyparium (a–p) and stalk (q–z) sclerites of *Paraminabea inflata* sp. nov. paratype (SCSTMBC240256). Scale bar, 10 μm.
**Figure S8:** Polyparium (a–n) and stalk (o–z) sclerites of *Paraminabea inflata* sp. nov. paratype (SCSTMBC240258). Scale bar, 10 μm.
**Figure S9:** Polyparium (a–m) and stalk (n–w) sclerites of *Paraminabea inflata* sp. nov. paratype (SCSTMBC240259). Scale bar, 10 μm.
**Figure S10:** Polyparium (a–r) and stalk (s–z) sclerites of *Paraminabea rubeusa* from Heizhou island in Zhuhai. Scale bar, 10 μm.

**Table S3:** Features of mitochondrial genomes used in phylogenetic analysis.

## Data Availability

The manuscript has been registered in ZooBank (https://zoobank.org/References/2bfee85b‐21a9‐41f3‐b908‐6bcf823d17d2). Type specimens of *Parasphaerasclera dimorpha* sp. nov. and *Paraminabea inflata* sp. nov. are deposited in the Tropical Marine Biodiversity Collections of the South China Sea, Chinese Academy of Sciences, Guangzhou, China. The *28S* rRNA sequences are available on GenBank under the accession numbers of PV617283–PV617286. The mitochondrial genome sequences and annotation are deposited in FigShare (https://doi.org/10.6084/m9.figshare.29107751.v1). The raw sequencing data in this study are deposited under BioProject PRJNA1260202 in GenBank.

## References

[ece372228-bib-0001] Au, A. C.‐S. , L. Zhang , S.‐S. Chung , and J.‐W. Qiu . 2014. “Diving Associated Coral Breakage in Hong Kong: Differential Susceptibility to Damage.” Marine Pollution Bulletin 85, no. 2: 789–796. 10.1016/j.marpolbul.2014.01.024. 24467858

[ece372228-bib-0002] Benayahu, Y. , and K. Fabricius . 2010. “Onsome Octocorallia (Alcyonacea) From Hong Kong, With Description of a New, *Paraminabea rubeusa* .” Pacific Science 64, no. 2: 285–296. 10.2984/64.2.285.

[ece372228-bib-0003] Benayahu, Y. , J. Ming‐Shiou , S. Perkol‐Finkel , and C.‐F. Dai . 2004. “Soft Corals (Octocorallia: Alcyonacea) From Southern Taiwan. II. Species Diversity and Distributional Patterns.” Zoological Studies 43, no. 3: 548–560.

[ece372228-bib-0004] Bernt, D. M. , and M. Middendorf . 2008. “Algorithm for Inferring Mitogenome Rearrangements in a Phylogenetic.” In Comparative Genomics, edited by C. E. Nelson and S. Vialette , 143. Springer. 10.1007/978-3-540-87989-3_11.

[ece372228-bib-0005] Binne Consultants Ltd . 1995. Marine Ecology of Hong Kong. Report on Underwater Dive Surveys (October 1991–November 1994). Geotechnical Engineering Office.

[ece372228-bib-0006] Bolger, A. M. , M. Lohse , and B. Usadel . 2014. “Trimmomatic: A Flexible Trimmer for Illumina Sequence Data.” Bioinformatics 30, no. 15: 2114–2120. 10.1093/BIOINFORMATICS/BTU170. 24695404 PMC4103590

[ece372228-bib-0007] Brockman, S. A. , and C. S. McFadden . 2012. “The Mitochondrial Genome of *Paraminabea aldersladei* (Cnidaria: Anthozoa: Octocorallia) Supports Intramolecular Recombination as the Primary Mechanism of Gene Rearrangement in Octocoral Mitochondrial Genomes.” Genome Biology and Evolution 4, no. 9: 994–1006. 10.1093/gbe/evs074. 22975720 PMC3468961

[ece372228-bib-0008] Bryce, M. , A. Poliseno , P. Alderslade , and S. Vargas . 2015. “Digitate and Capitate Soft Corals (Cnidaria: Octocorallia: Alcyoniidae) From Western Australia With Reports on New Species and New Australian Geographical Records.” Zootaxa 3963, no. 2: 160–200. 10.11646/zootaxa.3963.2.2. 26249397

[ece372228-bib-0009] Capella‐Gutiérrez, S. , J. M. Silla‐Martínez , and T. Gabaldón . 2009. “trimAl: A Tool for Automated Alignment Trimming in Large‐Scale Phylogenetic Analyses.” Bioinformatics 25, no. 15: 1972–1973. 10.1093/bioinformatics/btp348. 19505945 PMC2712344

[ece372228-bib-0061] Chan, A. L. K. , K. K. Chan , C. L. S. Choi , et al. 2005. “Field Guide to Hard Corals of Hong Kong.” Agriculture, Fisheries and Conservation Department, The Hong Kong SAR Government.

[ece372228-bib-0010] Chao, C.‐H. , Y.‐C. Wu , Z.‐H. Wen , and J.‐H. Sheu . 2013. “Steroidal Carboxylic Acids From Soft Coral *Paraminabea acronocephala* .” Marine Drugs 11, no. 1: 136–145. 10.3390/md11010136. 23344155 PMC3564163

[ece372228-bib-0011] Chung, S. , A. Au , and J.‐W. Qiu . 2013. “Understanding the Underwater Behaviour of Scuba Divers in Hong Kong.” Environmental Management 51: 824–837. 10.1007/s00267-013-0023-y. 23471632

[ece372228-bib-0012] Chung, T. H. , W. Dellisanti , K. P. Lai , J. Wu , J.‐W. Qiu , and L. L. Chan . 2024. “Local Conditions Modulated the Effects of Marine Heatwaves on Coral Bleaching in Subtropical Hong Kong Waters.” Coral Reefs 43: 1235–1247. 10.1007/s00338-024-02533-5.

[ece372228-bib-0013] Clark, T. H. 1997. “The Distribution of Ahermatypic Corals at Cape D'aguilar, Hong Kong.” In Proceedings, 8th International Marine Biology Workshop: The Marine Flora and Fauna of Hong Kong and Southern China, Hong Kong, 1995, 219–232. Hong Kong University Press.

[ece372228-bib-0014] Donath, A. , F. Jühling , M. Al‐Arab , et al. 2019. “Improved Annotation of Protein‐Coding Genes Boundaries in Metazoan Mitochondrial Genomes.” Nucleic Acids Research 47, no. 20: 10543–10552. 10.1093/NAR/GKZ833. 31584075 PMC6847864

[ece372228-bib-0015] Dumount, C. P. , D. C. C. Lau , J. C. Astudillo , K. F. Fong , S. T. C. Chak , and J.‐W. Qiu . 2013. “Coral Bioerosion by the Sea Urchin *Diadema setosum* in Hong Kong: Susceptibility of Different Coral Species.” Journal of Experimental Marine Biology and Ecology 441: 71–79. 10.1016/j.jembe.2013.01.018.

[ece372228-bib-0016] Fabricius, K. E. , and P. Alderslade . 2001. “Soft Corals and Sea Fans: A Comprehensive Guide to the Tropical Shallow Water Genera of the Central‐West Pacific, the Indian Ocean and the Red Sea.” https://www.waterbouwkundiglaboratorium.be/nl/publicaties/documentatiecentrum/catalogus.

[ece372228-bib-0017] Fabricius, K. E. , and D. McCorry . 2006. “Changes in Octocoral Communities and Benthic Cover Along a Water Quality Gradient in the Reefs of Hong Kong.” Marine Pollution Bulletin 52, no. 1: 22–33. 10.1016/j.marpolbul.2005.08.004. 16212989

[ece372228-bib-0019] Guindon, S. , J.‐F. Dufayard , V. Lefort , M. Anisimova , W. Hordijk , and O. Gascuel . 2010. “New Algorithms and Methods to Estimate Maximum‐Likelihood Phylogenies: Assessing the Performance of PhyML 3.0.” Systematic Biology 59, no. 3: 307–321. 10.1093/sysbio/syq010. 20525638

[ece372228-bib-0020] Hoang, D. T. , O. Chernomor , A. von Haeseler , B. Q. Minh , and L. S. Vinh . 2018. “UFBoot2: Improving the Ultrafast Bootstrap Approximation.” Molecular Biology and Evolution 35, no. 2: 518–522. 10.1093/MOLBEV/MSX281. 29077904 PMC5850222

[ece372228-bib-0022] Huang, D. , Y. L. Wilfredo , B. W. Hoeksema , et al. 2015. “Extraordinary Diversity of Reef Corals in the South China Sea.” Marine Biodiversity 45: 157–168. 10.1007/s12526-014-0236-1.

[ece372228-bib-0023] Kalyaanamoorthy, S. , B. Q. Minh , T. K. F. Wong , A. von Haeseler , and L. S. Jermiin . 2017. “ModelFinder: Fast Model Selection for Accurate Phylogenetic Estimates.” Nature Methods 14, no. 6: 587–589. 10.1038/nmeth.4285. 28481363 PMC5453245

[ece372228-bib-0024] Katoh, K. , K. Misawa , K. Kuma , and T. Miyata . 2002. “MAFFT: A Novel Method for Rapid Multiple Sequence Alignment Based on Fast Fourier Transform.” Nucleic Acids Research 30, no. 14: 3059–3066. 10.1093/NAR/GKF436. 12136088 PMC135756

[ece372228-bib-0025] Lam, K. , and B. Morton . 2008. “Soft Corals, Sea Fans, Gorgonians (Octocorallia: Alcyonacea) and Black and Wire Corals (Ceriantipatharia: Antipatharia) From Submarine Caves in Hong Kong With a Checklist of Local Species and a Description of a New Species of *Paraminabea* .” Journal of Natural History 42, no. 9–12: 749–780. 10.1080/00222930701862708.

[ece372228-bib-0026] Lam, K. , P. K. S. Shin , and P. Hodgson . 2007. “Severe Bioerosion Caused by an Outbreak of Corallivorous *drupella* and *Diadema* at Hoi ha Wan Marine Park, Hong Kong.” Coral Reefs 26: 893. 10.1007/s00338-007-0288-9.

[ece372228-bib-0027] Letunic, I. , and P. Bork . 2021. “Interactive Tree of Life (iTOL) v5: An Online Tool for Phylogenetic Tree Display and Annotation.” Nucleic Acids Research 49, no. W1: W293–W296. 10.1093/NAR/GKAB301. 33885785 PMC8265157

[ece372228-bib-0028] Li, C. 1986. “The Alcyonacea in Hong Kong Waters.” Tropic Oceanology 5: 19–25.

[ece372228-bib-0029] Maucieri, D. G. , and J. K. Baum . 2023. “Impacts of Heat Stress on Soft Corals, an Overlooked and Highly Vulnerable Component of Coral Reef Ecosystems, at a Central Equatorial Pacific Atoll.” Biological Conservation 262: 109328. 10.1016/j.biocon.2021.109328.

[ece372228-bib-0030] McCorry, D. 2002. “Hong Kong's Scleractinian Coral Communities: Status, Threats and Proposals for Management.” PhD Thesis, The Univerisity of Hong Kong, Hong Kong.

[ece372228-bib-0031] McFadden, C. S. , P. Alderslade , L. P. Van Ofwegen , H. Johnsen , and A. Rusmevichientong . 2006. “Phylogenetic Relationships Within the Tropical Soft Coral Genera *Sarcophyton* and *Lobophytum* (Anthozoa, Octocorallia).” Invertebrate Biology 125, no. 4: 288–305. 10.1111/j.1744-7410.2006.00070.x.

[ece372228-bib-0032] McFadden, C. S. , R. Cordeiro , K. Samimi‐Namin , G. Williams , and L. van Ofwegen . 2025a. “World List of Octocorallia. *Parasphaerasclera* McFadden & van Ofwegen, 2013.” World Register of Marine Species. https://www.marinespecies.org/aphia.php?p=taxdetails&id=743777.

[ece372228-bib-0033] McFadden, C. S. , R. Cordeiro , K. Samimi‐Namin , G. Williams , and L. van Ofwegen . 2025b. “World List of Octocorallia. *Paraminabea* Williams & Alderslade, 1999.” World Register of Marine Species. https://www.marinespecies.org/aphia.php?p=taxdetails&id=267691.

[ece372228-bib-0034] McFadden, C. S. , and L. van Ofwegen . 2013. “Molecular Phylogenetic Evidence Supports a New Family of Octocorals and a New Genus of Alcyoniidae (Octocorallia, Alcyonacea).” ZooKeys 346, no. 11: 59–83. 10.3897/zookeys.346.6270. PMC3821066 24223488

[ece372228-bib-0035] Minh, B. Q. , H. A. Schmidt , O. Chernomor , et al. 2020. “IQ‐TREE 2: New Models and Efficient Methods for Phylogenetic Inference in the Genomic Era.” Molecular Biology and Evolution 37, no. 5: 1530–1534. 10.1093/MOLBEV/MSAA015. 32011700 PMC7182206

[ece372228-bib-0036] Morton, B. , and G. Blackmore . 2009. “Seasonal Variations in the Density of and Corallivory by *Drupella Rugosa* and *Cronia Margariticola* (Caenogastropoda: Muricidae) From the Coastal Waters of Hong Kong: ‘Plagues’ or ‘Aggregations’.” Journal of the Marine Biological Association of the United Kingdom 89, no. 3: 147–159. 10.1093/mollus/eyx020.

[ece372228-bib-0038] Nurk, S. , A. Bankevich , D. Antipov , et al. 2013. “Assembling Genomes and Mini‐Metagenomes From Highly Chimeric Reads.” Lecture Notes in Computer Science (Including Subseries Lecture Notes in Artificial Intelligence and Lecture Notes in Bioinformatics) 7821: 158–170. 10.1007/978-3-642-37195-0_13.

[ece372228-bib-0039] Puillandre, N. , S. Brouillet , and G. Achaz . 2020. “ASAP: Assemble Species by Automatic Partitioning.” Molecular Ecology Resources 21, no. 2: 609–620. 10.1111/1755-0998.13281. 33058550

[ece372228-bib-0040] Qiu, J.‐W. , D. C. C. Lau , C.‐C. Cheang , and W.‐K. Chow . 2014. “Community‐Level Destruction of Hard Corals by the Sea Urchin *Diadema setosum* .” Marine Pollution Bulletin 85, no. 2: 783–788. 10.1016/j.marpolbul.2013.12.012. 24360335

[ece372228-bib-0041] Quattrini, A. M. , L. J. McCartin , E. E. Easton , et al. 2024. “Skimming Genomes for Systematics and DNA Barcodes of Corals.” Ecology and Evolution 14, no. 5: e11254. 10.1002/ece3.11254. 38746545 PMC11091489

[ece372228-bib-0042] Quattrini, A. M. , D. Morrissey , and L. J. Mccartin . 2025. “A New Soft Coral Species From the Gulf of Mexico (Octocorallia: Scleralcyonacea: Parasphaerascleridae).” Zootaxa 5601, no. 3: 545–557. 10.11646/zootaxa.5601.3.8. 40173685

[ece372228-bib-0043] Quattrini, A. M. , K. E. Snyder , R. Purow‐Ruderman , et al. 2023. “Mito‐Nuclear Discordance Within Anthozoa, With Notes on Unique Properties of Their Mitochondrial Genomes.” Scientific Reports 13, no. 1: 7443. 10.1038/s41598-023-34059-1. 37156831 PMC10167242

[ece372228-bib-0044] Quattrini, A. M. , T. Wu , K. Soong , M.‐S. Jeng , Y. Benayahu , and C. S. McFadden . 2019. “A Next Generation Approach to Species Delimitation Reveals the Role of Hybridization in a Cryptic Species Complex of Corals.” BMC Evolutionary Biology 19: 116. 10.1186/s12862-019-1427-y. 31170912 PMC6555025

[ece372228-bib-0045] Rambaut, A. , A. J. Drummond , D. Xie , G. Baele , and M. A. Suchard . 2018. “Posterior Summarization in Bayesian Phylogenetics Using Tracer 1.7.” Systematic Biology 67, no. 5: 901–904. 10.1093/SYSBIO/SYY032. 29718447 PMC6101584

[ece372228-bib-0046] Ronquist, F. , M. Teslenko , P. Van Der Mark , et al. 2012. “MrBayes 3.2: Efficient Bayesian Phylogenetic Inference and Model Choice Across a Large Model Space.” Systematic Biology 61, no. 3: 539–542. 10.1093/SYSBIO/SYS029. 22357727 PMC3329765

[ece372228-bib-0048] Tamura, K. , G. Stecher , and S. Kumar . 2021. “MEGA11: Molecular Evolutionary Genetics Analysis Version 11.” Molecular Biology and Evolution 38, no. 7: 3022–3027. 10.1093/MOLBEV/MSAB120. 33892491 PMC8233496

[ece372228-bib-0049] Tillich, M. , P. Lehwark , T. Pellizzer , et al. 2017. “GeSeq—Versatile and Accurate Annotation of Organelle Genomes.” Nucleic Acids Research 45, no. W1: W6–W11. 10.1093/NAR/GKX391. 28486635 PMC5570176

[ece372228-bib-0050] Williams, G. C. , and P. Alderslade . 1999. “Revisionary Systematics of the Western Pacific Soft Coral Genus *Minabea* (Octocorallia: Alcyoniidae), With Descriptions of a Related New Genus and Species From the Indo‐Pacific.” Proceedings of the California Academy of Sciences 51: 337–364.

[ece372228-bib-0063] Williams, G. , and C. Mattison . 2018. “Microscope Slide or SEM Stub Preparation for Octocoral Sclerites or Other Invertebrate Spicules.” https://research.calacademy.org/research/izg/OctoResearchTech.htm.

[ece372228-bib-0051] Wong, J. S. Y. , Y. K. S. Chan , C. S. L. Ng , K. P. P. Tun , E. S. Darling , and D. W. Huang . 2018. “Comparing Patterns of Taxonomic, Functional and Phylogenetic Diversity in Reef Coral Communities.” Coral Reefs 37: 737–750. 10.1007/s00338-018-1698-6.

[ece372228-bib-0052] Xie, J. Y. , J. C. Wong , C. P. Dumont , N. Goodkin , and J.‐W. Qiu . 2016. “Borehole Density on the Surface of Living Porites Corals as an Indicator of Sedimentation in Hong Kong.” Marine Pollution Bulletin 108, no. 1–2: 87–93. 10.1016/j.marpolbul.2016.04.055. 27179996

[ece372228-bib-0053] Xie, J. Y. , Y. H. Yeung , C. K. Kwok , et al. 2020. “Localized Bleaching and Quick Recovery in Hong Kong's Coral Communities.” Marine Pollution Bulletin 153: 110950. 10.1016/j.marpolbul.2020.110950. 32056854

[ece372228-bib-0054] Yeung, C. W. , C. C. Cheang , M. W. Lee , H. L. Fung , W. K. Chow , and P. Ang . 2014. “Environmental Variabilities and the Distribution of Octocorals and Black Corals in Hong Kong.” Marine Pollution Bulletin 85, no. 2: 774–782. 10.1016/j.marpolbul.2013.12.043. 24434001

[ece372228-bib-0055] Yeung, Y. H. , J. Y. Xie , C. K. Kwok , et al. 2021. “Hong Kong's Subtropical Scleractinian Coral Communities: Baseline, Environmental Drivers and Management Implications.” Marine Pollution Bulletin 167: 112289. 10.1016/j.marpolbul.2021.112289. 33773418

[ece372228-bib-0056] Yiu, S. K. F. , and J. W. Qiu . 2022. “Three New Species of the Sun Coral Genus *Tubastraea* (Scleractinia: Dendrophylliidae) From Hong Kong, China.” Zoological Studies 61: e45. 10.6620/ZS.2022.61-45. 36568806 PMC9745568

[ece372228-bib-0057] Zhang, D. , F. Gao , I. Jakovlić , et al. 2020. “PhyloSuite: An Integrated and Scalable Desktop Platform for Streamlined Molecular Sequence Data Management and Evolutionary Phylogenetics Studies.” Molecular Ecology Resources 20, no. 1: 348–355. 10.1111/1755-0998.13096. 31599058

[ece372228-bib-0062] Zhao, M.‐H. , J. C.‐H. Ip , C. M. How , Y.‐X. Li , D. Deconinck , and J.‐W. Qiu . 2024. “DNA Barcode Reference Database and Gap Analysis for Biomonitoring Hong Kong's Marine Animals.” Regional Studies in Marine Science 8: 103946. 10.1016/j.rsma.2024.103946.

[ece372228-bib-0058] Zhao, Y. , Y. S. Law , X. Zhai , K. Zhou , M. Chen , and J.‐W. Qiu . 2022. “Urban Coral Communities and Water Quality Parameters Along the Coasts of Guangdong Province, China.” Marine Pollution Bulletin 180: 113821. 10.1016/j.marpolbul.2022.113821. 35688066

[ece372228-bib-0059] Zheng, S. , P. Poczai , J. Hyvönen , J. Tang , and A. Amiryousefi . 2020. “Chloroplot: An Online Program for the Versatile Plotting of Organelle Genomes.” Frontiers in Genetics 11, no. 9: 1123. 10.3389/FGENE.2020.576124/BIBTEX. PMC7545089 33101394

[ece372228-bib-0060] Zou, R. L. , and P. J. B. Scott . 1982. “The Gorgonacea of Hong Kong.” In Proceedings, 1st International Marine Biology Workshop: The Marine Flora and Fauna of Hong Kong and Southern China, Hong Kong, 135–159. Hong Kong University Press.

