# Supplementary material for: Hidden in Plain Sight: Integrative Taxonomy Discovers Two New Species of Digitate Soft Corals in the Urban Waters of China's Greater Bay Area
Source: Ecol Evol. 2025 Sep 25;15(10):e72228. doi: 10.1002/ece3.72228 (PMC12463571; doi:10.1002/ece3.72228)
Supplement: Supplementary file 1 — Table S1: Data matrices and analysis approaches. Table S2: K2P distance analysis based on MutS gene fragment. Figure S1: Colonies of Paraminabea inflata sp. nov. (a, b) and Parasphaerasclera dimorpha sp. nov. (c, d). Scale bar, 10 mm. Figure S2: ASAP group results based on MutS dataset. Figure S3: Mitochondrial genome architecture of three soft corals. Figure S4: Phylogenetic analysis of soft corals using 14 mitochondrial coding genes (16,726 bp) and their gene order structure. This topology is supported by the ML analysis result. Square in node, fully supported by ML and BI analysis; Value in node, ultrabootstrap value/Bayesian Posterior Probability over 70/0.70; Ms.: MutS. Figure S5: Polyparium (a‐r) and stalk (s–z) sclerites of Parasphaesclera dimorpha sp. nov. paratype (SCSTMBC240254). Scale bar, 10 μm. Figure S6: Comparative morphological characteristics of Parasphaesclera dimorpha sp. nov. (a, c) and Parasphaesclera grayi (b, d). Figure S7: Polyparium (a–p) and stalk (q–z) sclerites of Paraminabea inflata sp. nov. paratype (SCSTMBC240256). Scale bar, 10 μm. Figure S8: Polyparium (a–n) and stalk (o–z) sclerites of Paraminabea inflata sp. nov. paratype (SCSTMBC240258). Scale bar, 10 μm. Figure S9: Polyparium (a–m) and stalk (n–w) sclerites of Paraminabea inflata sp. nov. paratype (SCSTMBC240259). Scale bar, 10 μm. Figure S10: Polyparium (a–r) and stalk (s–z) sclerites of Paraminabea rubeusa from Heizhou island in Zhuhai. Scale bar, 10 μm. [file ECE3-15-e72228-s001.docx]

**Supplementary Tables and Figures**

**Tables**

**Table S1 Data matrices and analysis approaches.**

**Table S2 K2P distance analysis based on *MutS* gene fragment**.

**Table S3 Features of mitochondrial genomes used in phylogenetic analysis.** (Supplementary File 2)

**Figures**

**Figure S1 Colonies of *Paraminabea inflata* sp. nov. (a-b) and *Parasphaerasclera dimorpha* sp. nov. (c-d).** Scale bar, 10 mm.

**Figure S2 ASAP group results based on *MutS* dataset.**

**Figure S3 Mitochondrial genome architecture of three soft corals.**

**Figure S4 Phylogenetic analysis of soft corals using 14 mitochondrial coding genes (16726 bp) and their gene order structure.** This topology is supported by the ML analysis result. Square in node, fully supported by ML and BI analysis; Value in node, ultrabootstrap value/ Bayesian Posterior Probability over 70/0.70; Ms: *MutS*.

**Figure S5 Polyparium (a-r) and stalk (s-z) sclerites of *Parasphaesclera dimorpha* sp. nov. paratype (SCSTMBC240254).** Scale bar, 10 μm.

**Figure S6 Comparative morphological characteristics of *Parasphaesclera dimorpha* sp. nov. (a, c) and *Parasphaesclera grayi* (b, d)*.***

**Figure S7 Polyparium (a-p) and stalk (q-z) sclerites of *Paraminabea inflata* sp. nov. paratype (SCSTMBC240256).** Scale bar, 10 μm.

**Figure S8 Polyparium (a-n) and stalk (o-z) sclerites of *Paraminabea inflata* sp. nov. paratype (SCSTMBC240258).** Scale bar, 10 μm.

**Figure S9 Polyparium (a-m) and stalk (n-w) sclerites of *Paraminabea inflata* sp. nov. paratype (SCSTMBC240259).** Scale bar, 10 μm.

**Figure S10 Polyparium (a-r) and stalk (s-z) sclerites of *Paraminabea rubeusa* from Heizhou island in Zhuhai.** Scale bar, 10 μm.

**Table S1**

| Matrix | data | Length (bp) | Informative sites (bp) | Best-fit model |
| --- | --- | --- | --- | --- |
| 1 | *MutS* | 2949 | 306 | GTR+F+G4 |
| 2 | *28S* | 795 | 131 | HKY+F+G4 |
| 3 | 14-mt-gene | 16726 | 4281 | GTR+F+I+G4 (mixed) |

**Table S2**

|  | A_d | Pm_a | Pm_r | Pm_i | Ps_m | Ps_g | Ps_d | Ps_a | Ps_k | Ps_r | Ps_v | Ps_s |
| --- | --- | --- | --- | --- | --- | --- | --- | --- | --- | --- | --- | --- |
| *A. digitatum* | NA |  |  |  |  |  |  |  |  |  |  |  |
| *Param. aldersladei* | 15.37 | 0.00 |  |  |  |  |  |  |  |  |  |  |
| *Param. rubeusa* | 16.05 | **0.88** | 0.00 |  |  |  |  |  |  |  |  |  |
| ***Param. inflata*** | 14.23 | **1.24** | **1.77** | NA |  |  |  |  |  |  |  |  |
| *Paras_mcfaddenae* | 13.14 | 7.22 | 8.22 | 6.63 | NA |  |  |  |  |  |  |  |
| *Paras. grayi* | 12.59 | 7.43 | 8.42 | 6.44 | 1.41 | **2.00** |  |  |  |  |  |  |
| ***Paras_dimorpha*** | 12.69 | 8.02 | 9.03 | 7.02 | 1.41 | **1.24** | NA |  |  |  |  |  |
| *Paras. aurea* | 13.36 | 7.82 | 8.83 | 6.83 | 1.59 | **1.24** | **0.88** | 0.00 |  |  |  |  |
| *Paras. kimberleyensis* | 12.27 | 7.43 | 8.43 | 6.44 | 1.23 | 0.89 | 1.59 | 1.77 | NA |  |  |  |
| *Paras. rotifera* | 12.40 | 7.52 | 8.52 | 6.53 | 2.96 | 3.33 | 3.33 | 3.51 | 3.15 | NA |  |  |
| *Paras. valdiviae* | 12.93 | 8.62 | 9.64 | 7.61 | 4.54 | 4.36 | 4.54 | 4.64 | 4.36 | 4.26 | 0.00 |  |
| *Paras_*sp. | 12.27 | 7.43 | 8.43 | 6.44 | 1.23 | 0.89 | 1.59 | 1.77 | 0.00 | 3.15 | 4.36 | NA |

**Figures**

**Figure S1**

**
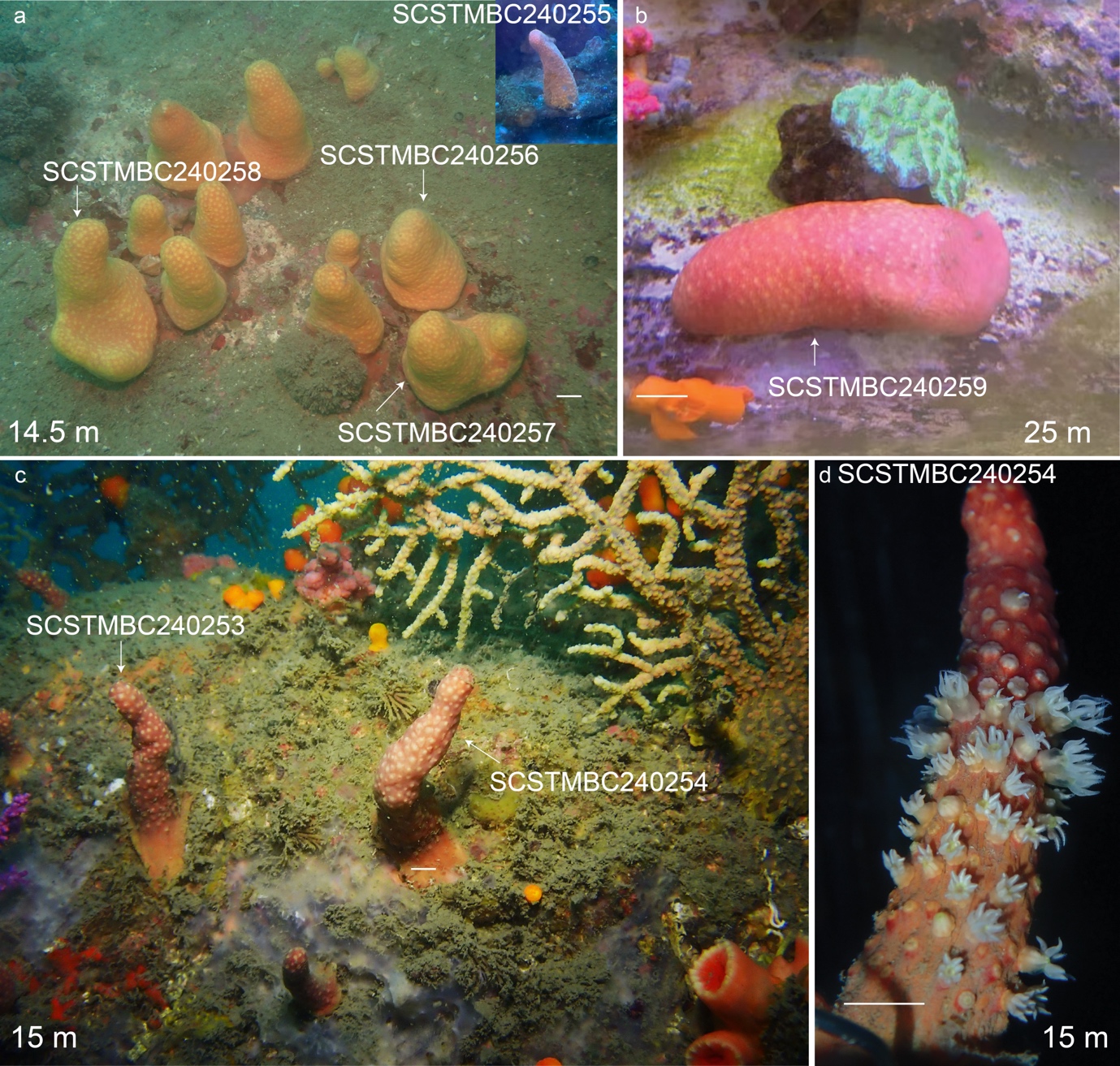
**

**Figure S2**

**
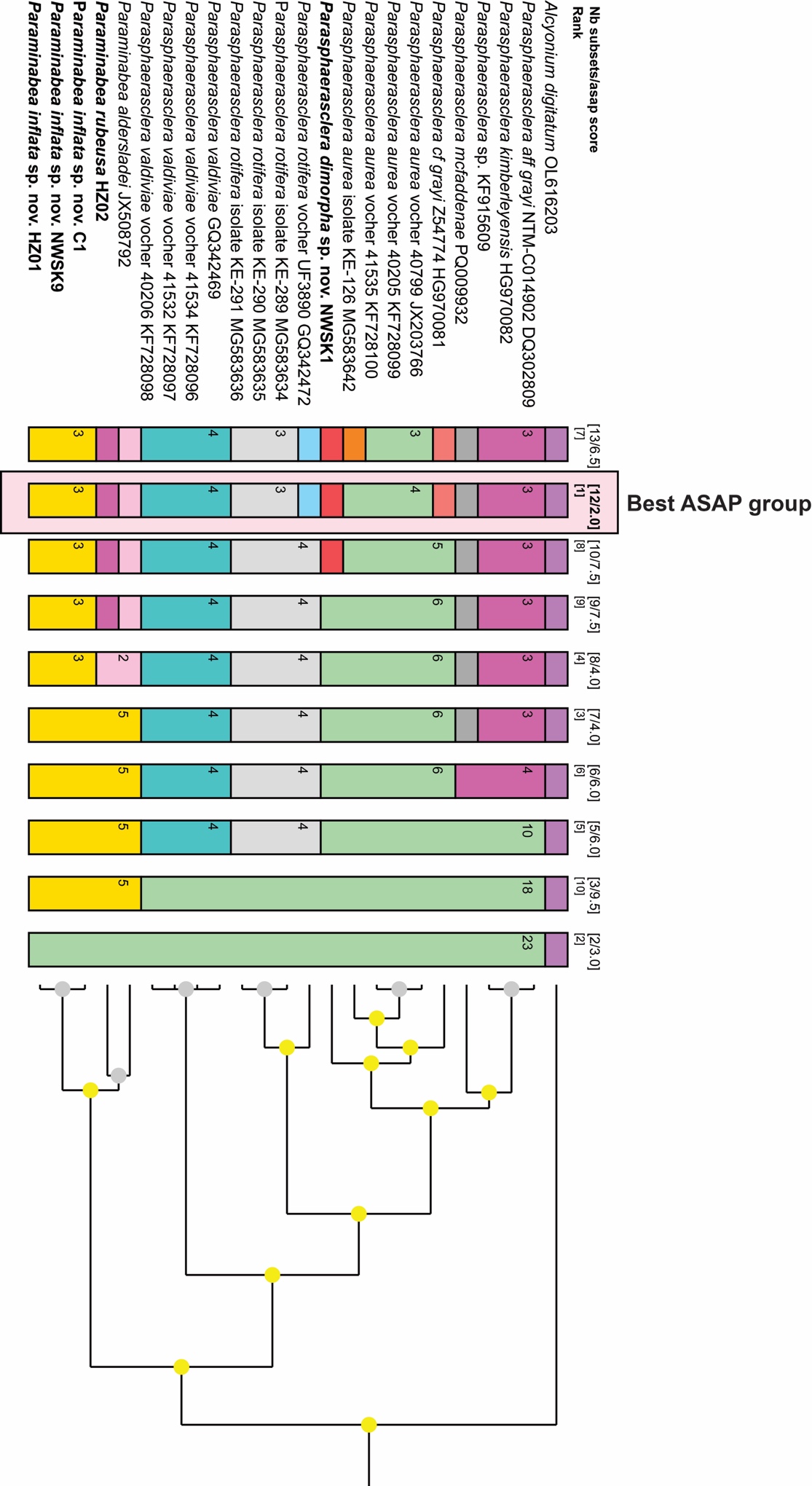
**

**Figure S3**

**
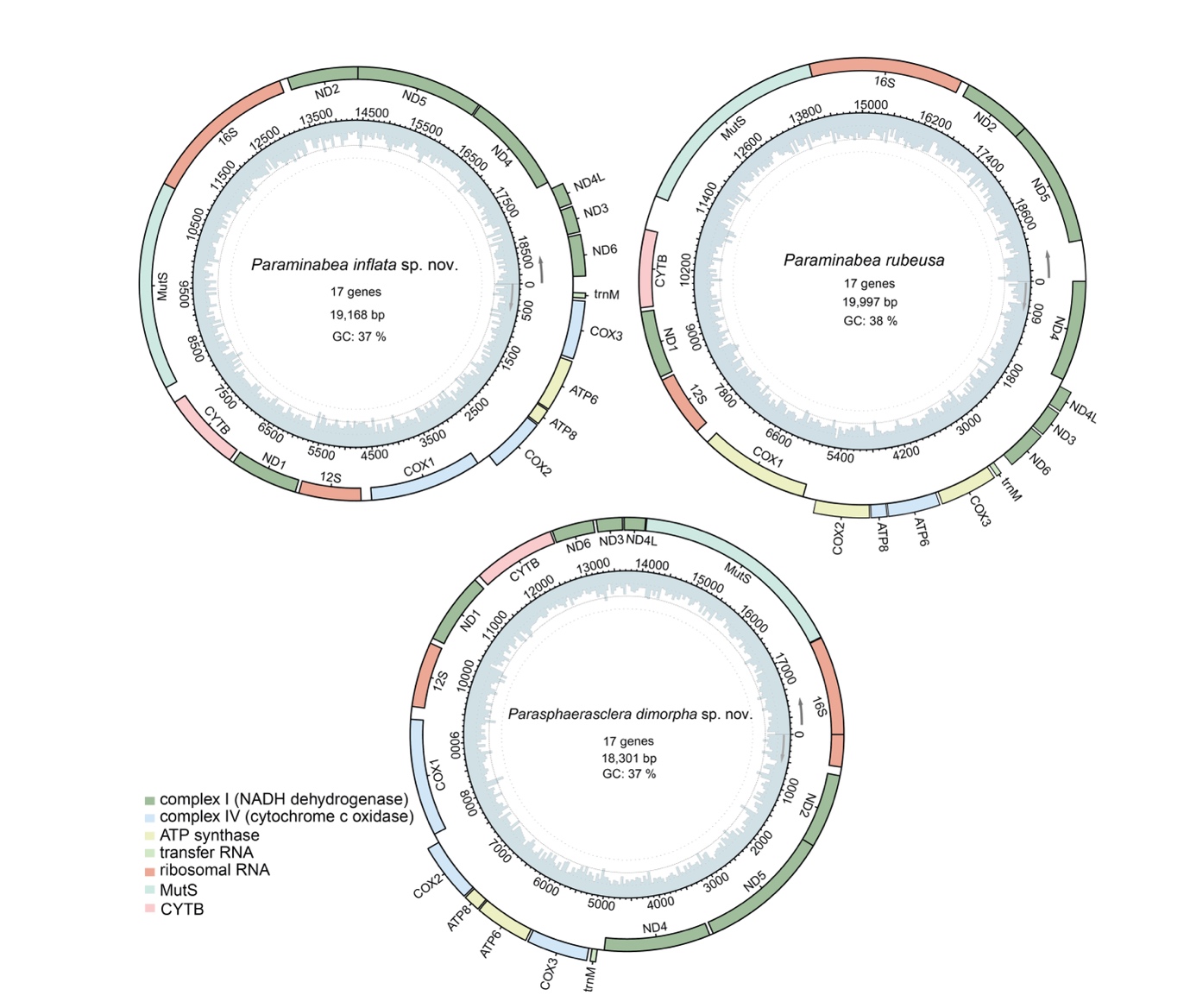
**

**Figure S4**


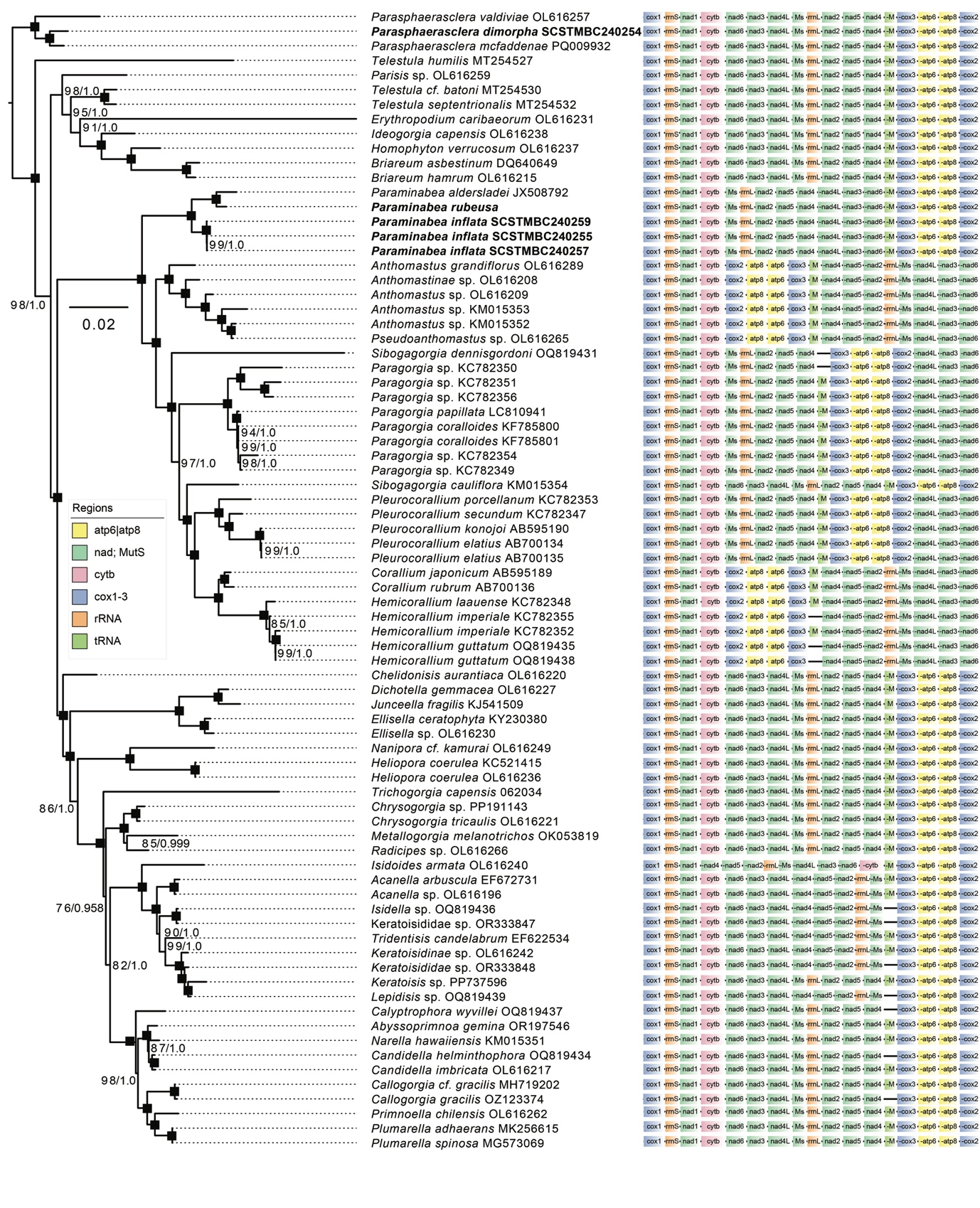


**Figure S5**

**
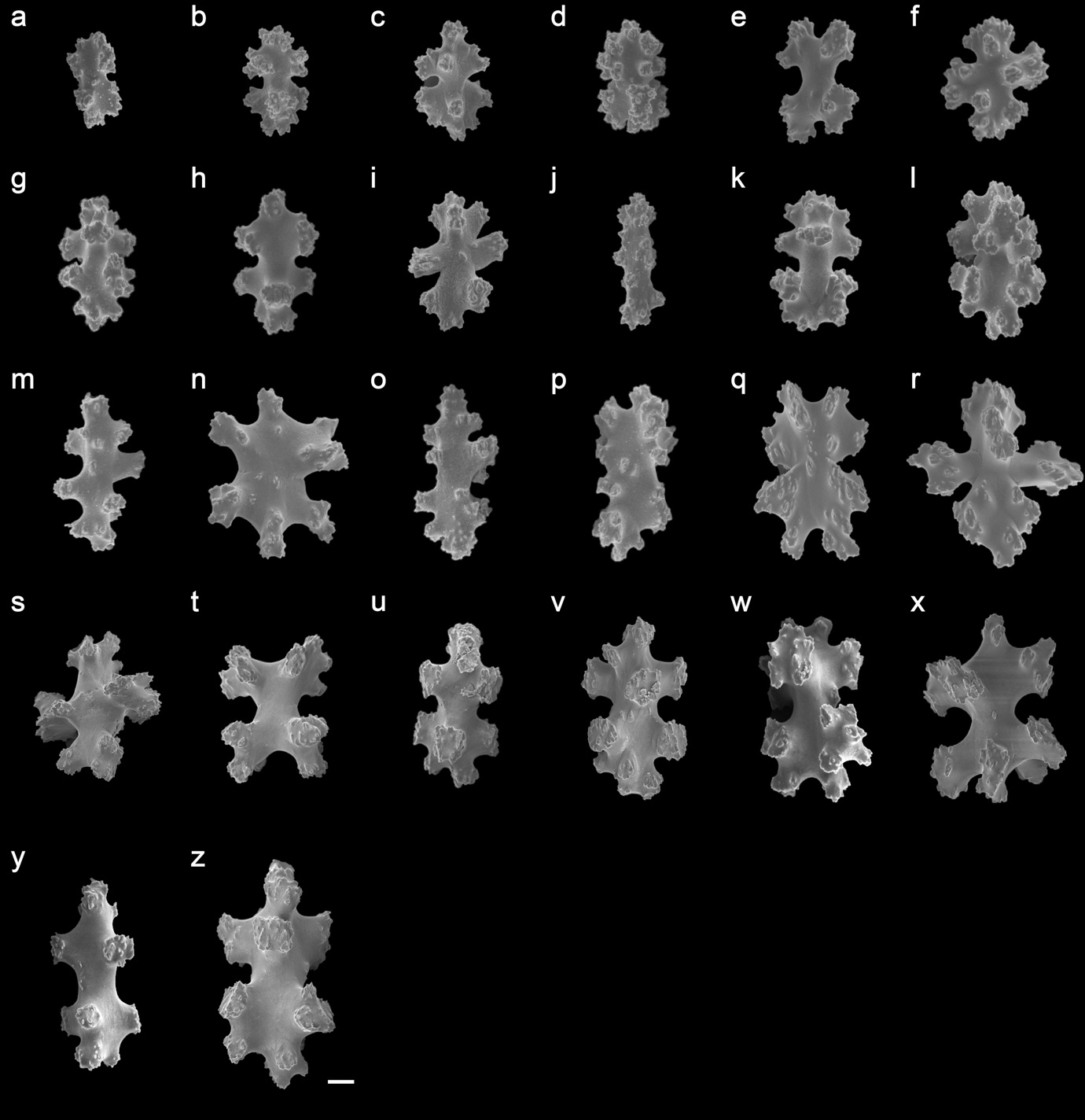
**

**Figure S6**

**
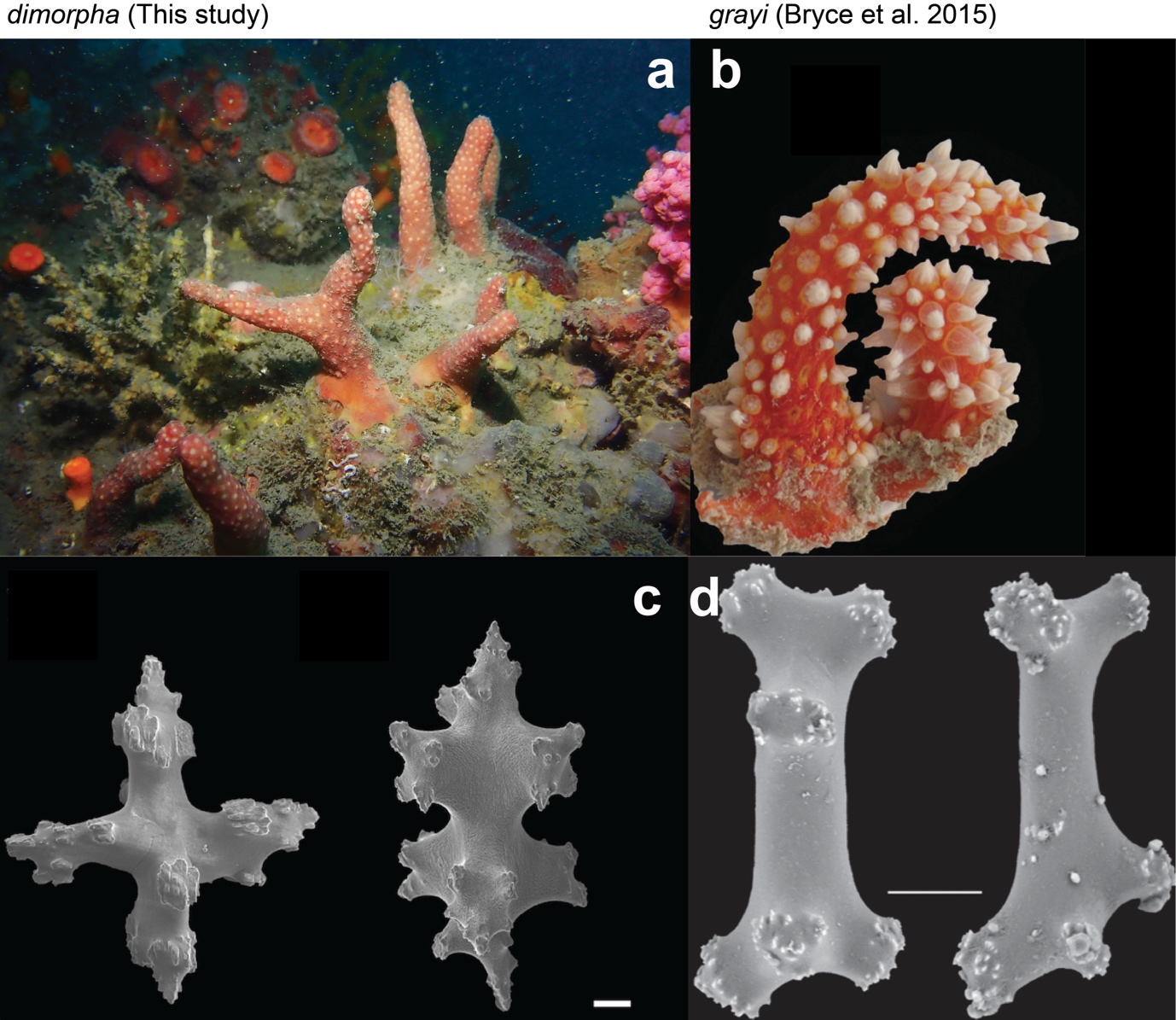
**

**Figure S7**

**
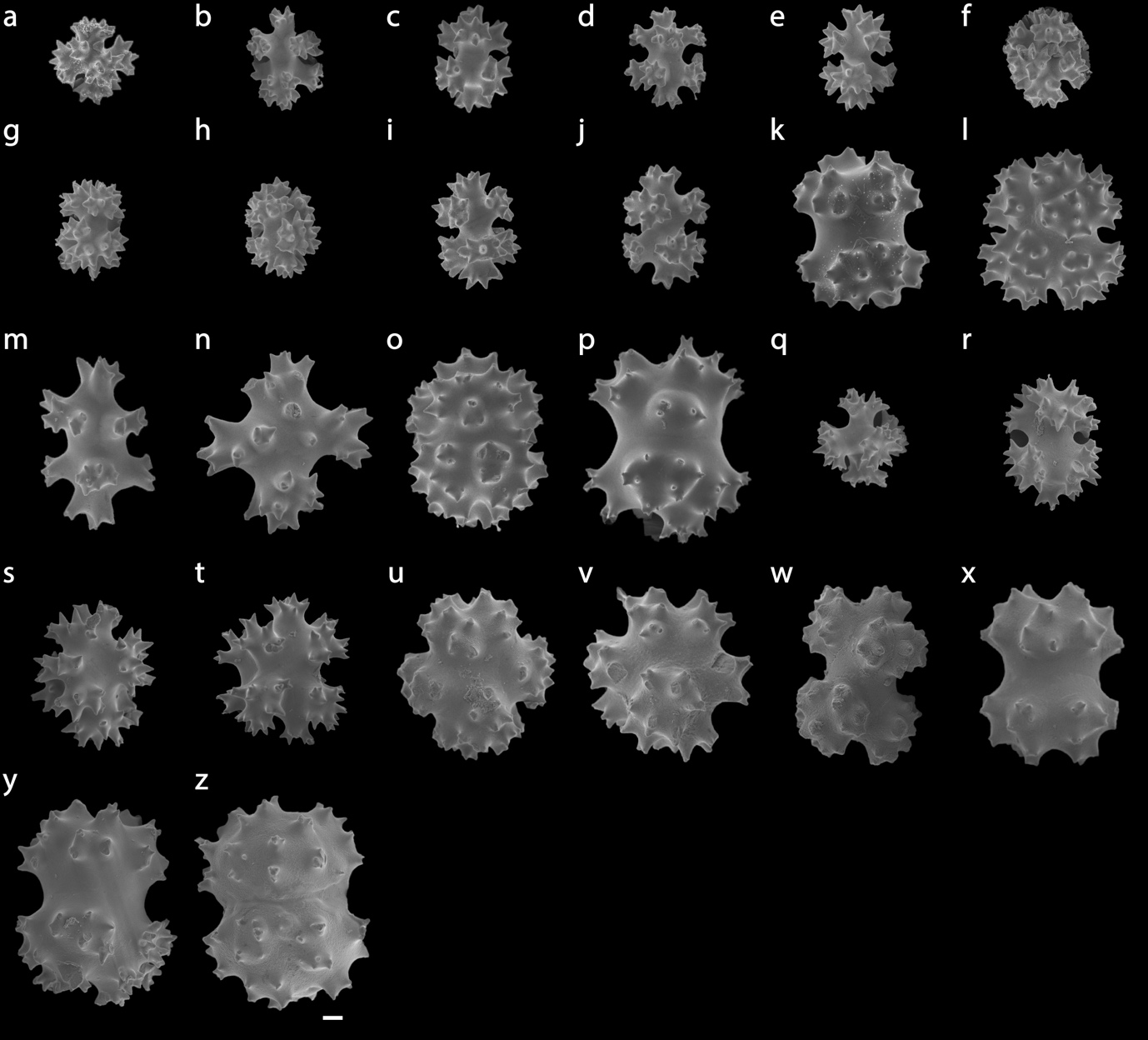
**

**Figure S8**

**
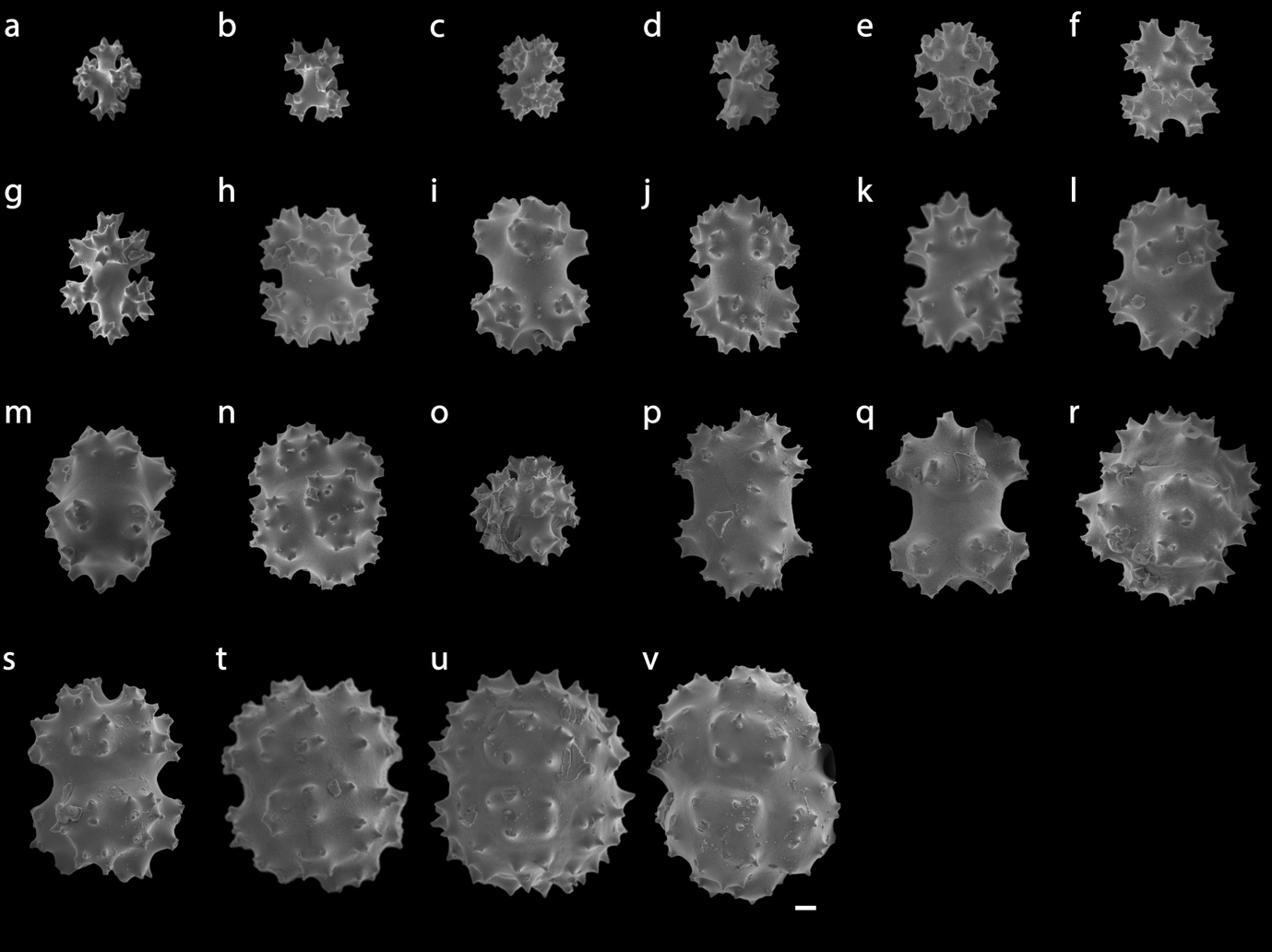
**

**Figure S9**

**
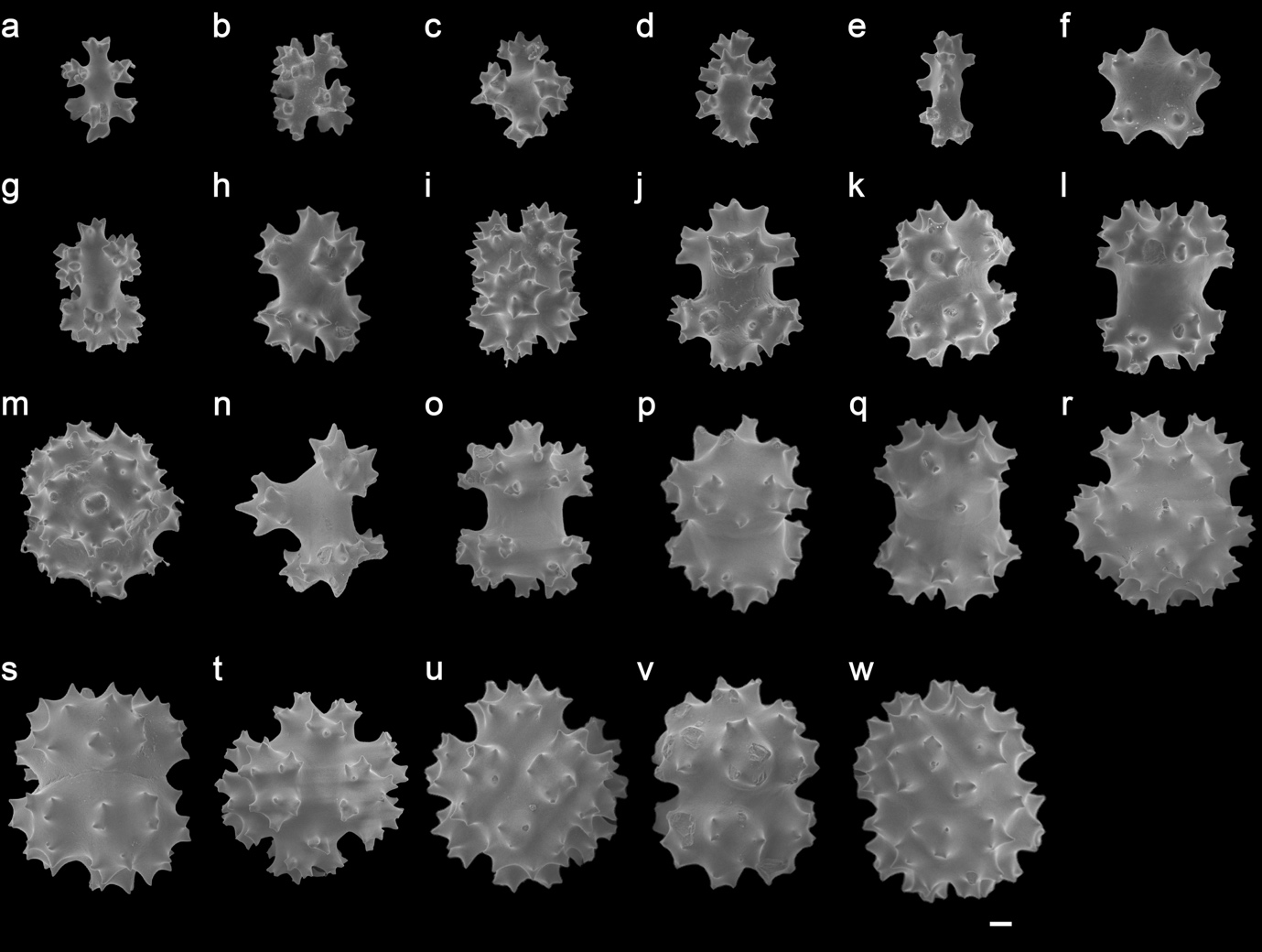
Figure S10**

**
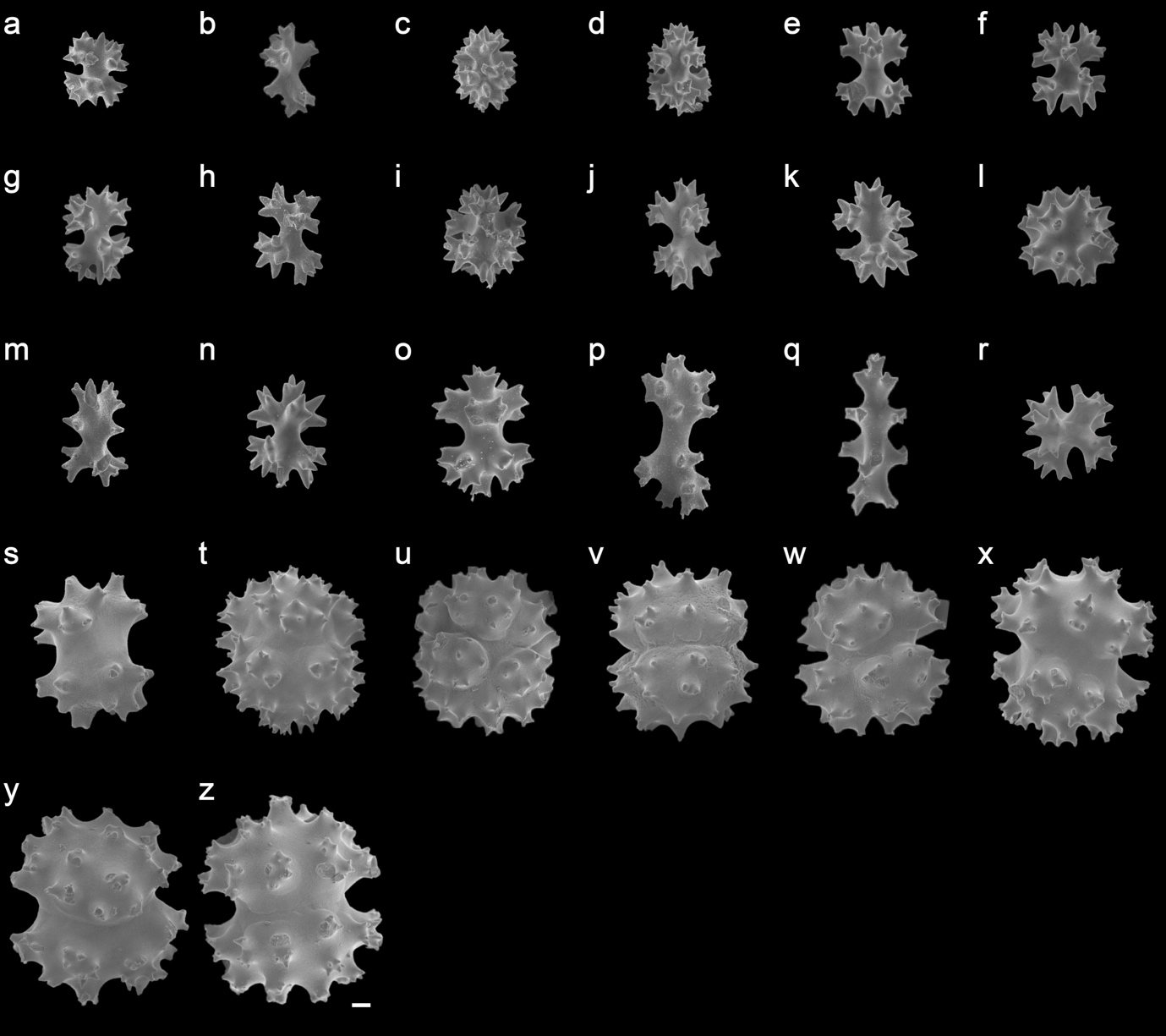
**
